# Reducing dynamic disorder in small-molecule organic semiconductors by suppressing large-amplitude thermal motions

**DOI:** 10.1038/ncomms10736

**Published:** 2016-02-22

**Authors:** Steffen Illig, Alexander S. Eggeman, Alessandro Troisi, Lang Jiang, Chris Warwick, Mark Nikolka, Guillaume Schweicher, Stephen G. Yeates, Yves Henri Geerts, John E. Anthony, Henning Sirringhaus

**Affiliations:** 1 Optoelectronics Group, Cavendish Laboratory, University of Cambridge, JJ Thompson Avenue, Madingley Road, Cambridge CB3 0HE, UK; 2 Department of Materials Science and Metallurgy, University of Cambridge, Charles Babbage Road, Cambridge CB3 0FS, UK; 3 Department of Chemistry and Centre for Scientific Computing, University of Warwick, Gibbet Hill Road, Coventry CV4 7AL, UK; 4 School of Chemistry, University of Manchester, Oxford Road, Manchester M13 0PL, UK; 5 Faculté des Sciences, Université Libre de Bruxelles, Boulevard du Triomphe, Brussels 1050, Belgium; 6 Department of Chemistry, University of Kentucky, Lexington, Kentucky 40506-005, USA

## Abstract

Thermal vibrations and the dynamic disorder they create can detrimentally affect the transport properties of van der Waals bonded molecular semiconductors. The low-energy nature of these vibrations makes it difficult to access them experimentally, which is why we still lack clear molecular design rules to control and reduce dynamic disorder. In this study we discuss the promising organic semiconductors rubrene, 2,7-dioctyl[1]benzothieno[3,2-b][1]benzothio-phene and 2,9-di-decyl-dinaphtho-[2,3-b:20,30-f]-thieno-[3,2-b]-thiophene in terms of an exceptionally low degree of dynamic disorder. In particular, we analyse diffuse scattering in transmission electron microscopy, to show that small molecules that have their side chains attached along the long axis of their conjugated core are better encapsulated in their crystal structure, which helps reduce large-amplitude thermal motions. Our work provides a general strategy for the design of new classes of very high mobility organic semiconductors with a low degree of dynamic disorder.

## Abstract (web summary)

Thermal vibration is harmful to charge transport in molecular semiconductors, which hinders the use of these materials in flexible electronics. Here, Illig *et al.* show that the vibration is suppressed when molecular side chains are attached to the long axis of conjugated cores.

## Graphical abstract

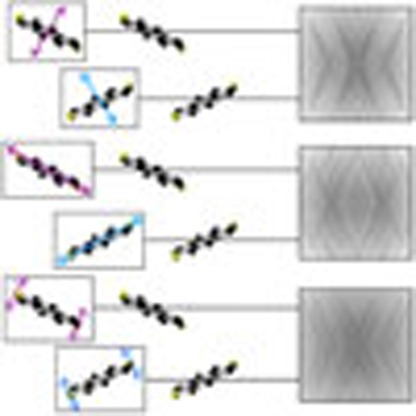

The capability of charge carriers to form delocalized electronic states is crucial to achieving organic semiconductors with high carrier mobilities >1–10 cm^2^ V^−1^ s^−1^. Charge delocalization is favoured by large transfer integrals, small electron–phonon coupling and the absence of energetic disorder. It is therefore not surprising that delocalized transport has been studied first in highly ordered systems such as organic crystals composed of small molecules. Both experimentally and theoretically, it has been shown that charge carriers in these systems can in fact be delocalized over a small number of molecules (typically on the order of 10), resulting in narrow bands of spatially extended electronic states that allow for a far more efficient charge transport mechanism than charge hopping between molecularly localized orbitals^1^.

The first step towards a high-mobility organic semiconductor is to start with a system that has an intrinsically high transfer integral *J*, allowing the charge carrier to form delocalized states^2^. It has been demonstrated that the crystal structure can be engineered during film deposition, sometimes enabling higher transfer integrals than in the equilibrium structure^3^. The second step is to eliminate static disorder such as chemical impurities or crystal defects, which often account for deep trap states^4^. In addition, in thin-film devices it is necessary to optimize charge injection and to avoid charge carrier trapping at the interfaces to obtain high-mobility systems^5^. It is believed that in the highest mobility systems available to date, device optimization has been pushed to an extent that wave function delocalization is not limited any more by static charge carrier traps but by thermal fluctuations in the transfer integrals, which is referred to as dynamic disorder^6^,^7^. An increasing number of high-performance organic semiconductors have been reported in the literature to exhibit a band-like temperature dependence of the mobility near room temperature^8^,^9^. This indicates that dynamic disorder is already one of the major factors limiting charge transport. A natural strategy to improve the performance of molecular semiconductors is therefore to reduce dynamic disorder.

One possibility to achieve such a reduction is by lowering the sensitivity of the transfer integrals to these vibrations, that is, by weakening the electron–phonon coupling^10^. We argue in this study that rubrene is an important representative of this approach; the high mobilities observed in single crystals^11^ can be rationalized with an exceptionally weak electron–phonon coupling, that is, the transfer integrals are insensitive to thermal fluctuations^12^. More fundamentally, dynamic disorder can be reduced by increasing the force constants between molecules, which helps to avoid large-amplitude thermal vibrations in the first place^10^.

As a result of the weak interactions between molecules, intermolecular displacements measure up to 0.5 Å at room temperature^6^,^13^. Such displacements Δ**r**=**r**_*i*_−**r**_*j*_ modulate the corresponding transfer integral *J*_*ij*_ to the same order of magnitude as the transfer integral itself^14^. The time scale of charge delocalization *t*_del_=10 fs is ∼100 times shorter than that of intermolecular vibrations *t*_int_=1 ps. This implies that charge carriers are transiently localized in dynamically generated molecular configurations with small transfer integrals^1^. The period of the intermolecular vibration is hereby crucial and has been found to be inversely proportional to the charge carrier mobility, that is, low-frequency, large-amplitude vibrations are most harmful for charge transport. The charge carrier mobility can therefore be improved by increasing the force constants between molecules^10^.

Our motivation for the present work is to investigate experimentally what molecular design strategies might be effective to reduce large-amplitude motions in molecular crystals. For this, we use a recently developed transmission electron microscopy (TEM) technique to characterize large-amplitude thermal vibrations in several classes of state-of-the-art, high-mobility crystalline organic semiconductors. We demonstrate that amplitudes of intermolecular vibrations can be reduced if the side chains are attached to the axis of the molecule's highest amplitude vibration. We will draw special attention to 2,7-dioctyl[1]benzothieno[3,2–b][1]benzothio-phene (C8-BTBT) and 2,9-di-decyl-dinaphtho-[2,3-b:20,30-f]-thieno-[3,2–b]-thiophene (C10-DNTT). These materials are ideal representatives of this molecular design class and have recently gained strong attention after high field-effect motilities were reported^15^,^16^. Our results explain the beneficial properties found in these materials in terms of an exceptionally small dynamic disorder.

## Results

### Brick-wall stacked crystals

**Figure 1:**
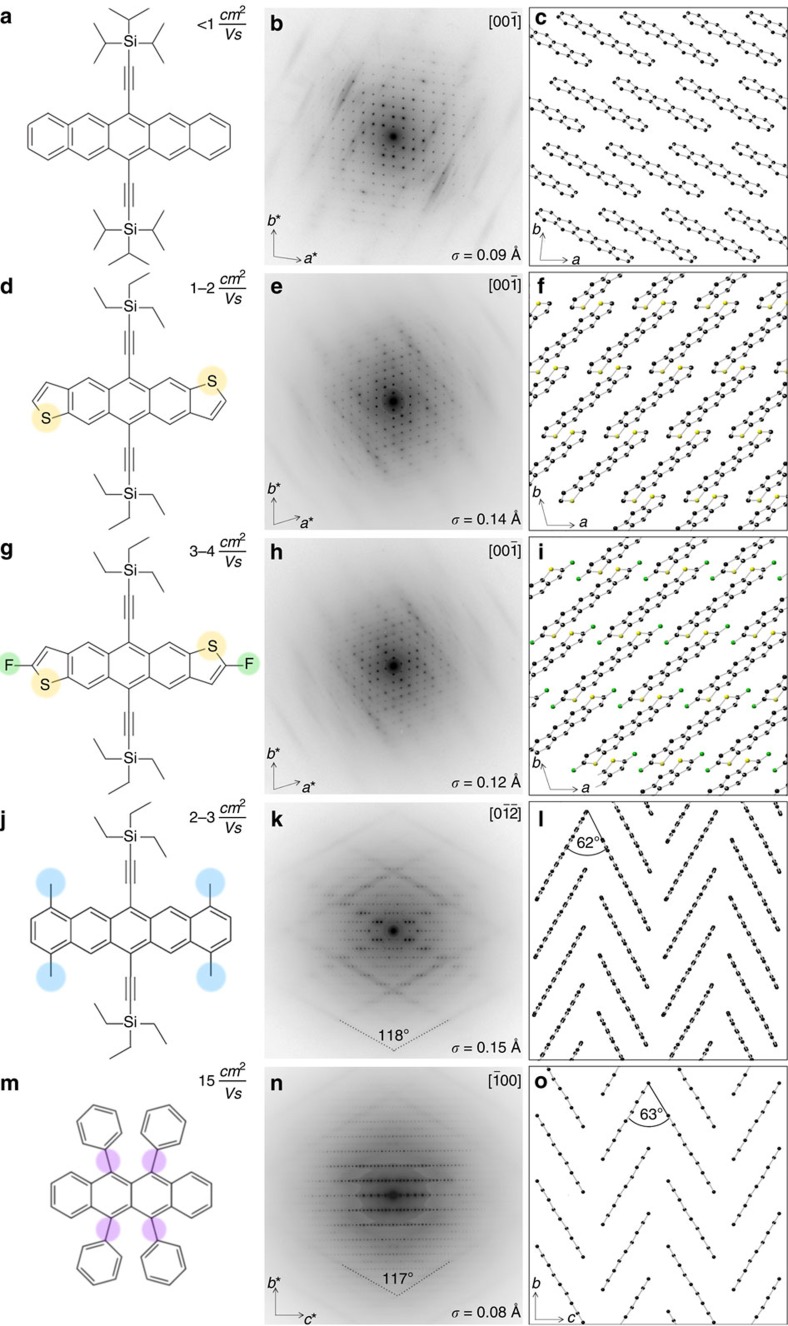
Molecular structure and electron diffraction pattern with corresponding crystal structure of different molecules. (**a**–**c**) TIPS-P, (**d**–**f**) TESADT, (**g**–**i**) diF-TESADT, (**j**–**l**) TMTES-P and (**m**–**o**) rubrene. The presented field-effect mobilities have been measured in single crystals that have been grown in the same condition than those studied by electron diffraction. These values are indicative only for these materials and higher values might be achieved if crystal growth and device fabrication was optimized further. Details are presented in the Supplementary Methods. Diffraction patterns are presented on a logarithmic intensity scale, which makes the faint streaking visible alongside the much stronger Bragg reflections. Refinement against simulated diffraction pattern yielded the amplitudes *σ* of the dominant long-axis vibration at room temperature. Crystal structures are oriented to correspond to the direction of the diffraction pattern. Hydrogens and side chains are omitted for clarity.

Our recently developed TEM technique provides a quantitative, experimental determination of the amplitude of intermolecular vibrations. The technique exploits the fact that large-amplitude thermal vibrations interrupt the periodicity of the crystal, resulting in incoherent scattering between Bragg reflections (Fig. 1). Rather than a uniform, diffuse background as expected from random atomic motion, vibrations in organic crystals create well-defined sheets or rods in reciprocal space. Such diffuse features are a result of correlated atomic motion^17^: strong intramolecular forces bind atoms of the molecular cores to relatively rigid units, moving within a weak intermolecular potential. Being a fingerprint of thermal vibrations, the diffuse features clearly weaken at low temperature measurements^13^. The electrons in our TEM pass through the organic thin film at ∼80% of the speed of light after being accelerated by a 300 kV potential^18^. At that velocity, every electron that contributes to the diffraction pattern encounters a static snapshot of the thermal crystal lattice, which typically vibrates with frequencies of 10^12^–10^13^ Hz (ref. 19). It is possible to simulate the diffraction pattern in the same way by generating a crystal that incorporates thermal vibrations as static displacements. Comparison and refinement of the simulated against the experimental diffraction pattern allows identifying the nature of the thermal vibrations that create the largest displacements^13^.

By applying this technique, it has been reported in ref. 13 that the dominant thermal motion in 6,13-bis(triisopropylsilylethynyl) pentacene (TIPS-P) corresponds to a translational vibration of the pentacene fragments in direction of their long axes (Fig. 1a–c). The side chains have been reported to not follow that vibration, a result that was confirmed in this work (Supplementary Fig. 1). This is rationalized with the clear steric separation between the molecular cores and the enmeshed planes formed by the hydrogen-rich side chains. Quantitative refinement of the simulated against the experimental diffraction patterns allowed estimating the amplitude *σ*=0.09 Å of the dominant thermal vibration in TIPS-P at room temperature (Fig. 1b). Here, amplitude refers to the standard deviation *σ* of the Gaussian that describes the distribution of thermal displacements at any moment. It is noteworthy that actual molecular displacements are therefore larger with ≈5% of displacements exceeding 2*σ* and intermolecular displacements Δ*r* between adjacent molecules being the sum of individual molecular displacements.

Here we investigate other molecular crystals with a similar ‘brick-wall'-type stacking, such as 5,11-bis(triethylsilylethynyl) anthradithiophene (TESADT) and its fluorinated analogue (diF-TESADT), shown in Fig. 1d–i. The crystal structures for these materials were taken from refs 20 and 21, respectively. Both structures could be verified by electron diffraction and have proven to be a good description for the investigated thin films. With these molecules it has been speculated that interactions between adjacent sulfur (yellow) and fluorine (green) atoms might better link neighbouring molecules and thus reduce thermal vibrations^22^. However, analysis of the diffraction data for these materials shows exactly as in TIPS-P a clear signature of strong intermolecular vibrations of the conjugated cores. Refinement against the experimental diffraction data revealed that the amplitude of the long-axis vibration is even larger than in TIPS-P (Fig. 1e,h). Therefore, the sulfur or fluorine substitution seems to have no significant effect in modulating the amplitude of thermal vibrations in TESADT and diF-TESADT. We note that our TESADT and diF-TESADT samples comprise a mixture of syn- and anti-conformers. Whether in pure syn- or anti-conformers the sulfur or fluorine interaction could be enhanced is currently under investigation^23^,^24^.

1,4,8,11-Tetramethyl-6,13-triethylsilylethynylpentacene (TMTES-P) shown in Fig. 1j–l has a similar molecular design than TIPS-P with additional methyl groups (blue) attached to the pentacene core. In contrast to TESADT and diF-TESADT, electron diffraction patterns taken from TMTES-P thin films were not consistent with the structure, reported in ref. 25 (Supplementary Figs 2 and 3). Instead, diffraction studies revealed that thin films of TMTES-P stack in the herringbone motif shown in Fig. 1l. This structure has been made available through the Cambridge Structural Database^26^. The diffuse scattering in these films is dominated by well-defined continuous streaks. Analysis shows that as for the previous materials, the diffuse streaks are a result of a strong translational motion of the conjugated cores in their long-axis direction. The herringbone structure with an angle of 62° between adjacent molecules results in two streaks at an angle of 180°−62°=118° compared with a single streaking direction in materials with only one molecular orientation. Simulation and refinement suggests again that neither the additional methyl groups that have been attached to the molecule (blue) nor its herringbone packing motif induce any reduction of the dominant long-axis vibration.

Rubrene (Fig. 1m–o) stacks in a similar crystal structure than TMTES-P thin films and has been extensively studied after high mobilities of>10 cm^2^ V^−1^ s^−1^ have been reported^11^,^27^. The structure of rubrene has been taken from ref. 28 and found to be in excellent agreement with our diffraction data shown in Fig. 1n. The diffuse scattering is strongly reminiscent of that in TMTES-P with well-defined continuous streaks that form an angle of 117°, in perfect agreement with the 63° angle between molecules in their herringbone structure. As for all materials presented in Fig. 1, thermal vibrations are strongly dominated by long-axis displacements of the molecular cores. Quantitative refinement resulted in a long-axis amplitude of *σ*=0.08 Å, which is at the lower end of the calculated amplitudes but still within a factor of 2 in comparison with the largest amplitude found in TMTES-P. Details of the simulations are described in Supplementary Note 1 and quantitative refinements are presented in Supplementary Figs 4–7.

Figure 1 shows typical mobility values measured in field effect transistors based on single crystal devices. The quoted values are meant to be indicative only; higher values might be achieved through optimization of crystal growth and device fabrication. However, it is clear that TIPS-P, TMTES-P, TESADT and diF-TESADT exhibit typical mobilities in the range of 1–5 cm^2^ V^−1^ s^−1^, whereas rubrene exhibits higher mobilities, in agreement with previous reports^9^. Details about single crystal device fabrication are provided in the Supplementary Methods; film and device characteristics are shown in Supplementary Figs 8–11.

### Rubrene

With respect to the identified long-axis vibration in these materials, the high mobilities reported in rubrene might be surprising. However, it is important to note that no direct conclusion regarding the electrical performance of a material can be drawn from the amplitudes of thermal vibrations alone. Even a very rigid material with negligible amplitudes will be a bad semiconductor if the transfer integrals are intrinsically low. It is rather believed that a high-mobility material requires a combination of high transfer integrals and low degree of dynamic disorder that does not compromise the electronic coupling^10^. Given the similarity between the rubrene and TMTES-P crystal structures, we compared the nearest-neighbour transfer integrals, computed with the same method given in ref. 6. We were surprised to find all three symmetry-independent nearest-neighbour transfer integrals in rubrene (1,135, 172 and 172 cm^−1^) to be lower than those in TMTES-P (2,082, 217 and 221 cm^−1^). This difference can be explained as a result of the larger *π*–*π* stacking distance in rubrene (3.74 Å) compared with TMTES-P (3.39 Å) but contradicts the higher mobilities in rubrene. Thus, even though the transfer integrals in rubrene single crystals are comparable to other high-performance organic semiconductors^27^, they do not provide an explanation of its remarkable mobility. A lower degree of dynamic disorder due to the slightly lower amplitude of the long-axis vibration might be a contributing factor; however, given the high-performance of rubrene single crystals we think there is another mechanism involved. da Silva Filho *et al.*^27^ have computed the evolution of the dominant transfer integral in rubrene as a function of long-axis displacement between adjacent molecules (Fig. 4 in ref. 27). The transfer integral falls on a local extrema, indicating that small intermolecular displacements imply only marginal changes as the slope of the transfer integral function is small close to its extremum, that is, the electron–phonon coupling is small. Both the low sensitivity of the transfer integral to intermolecular displacements and the reduced amplitude in comparison with TMTES-P are likely to contribute to a much lower degree of dynamic disorder in rubrene. This allows explaining the better performance of rubrene compared with TMTES-P despite its smaller transfer integrals. We therefore suggest that the good performance of rubrene single crystals is at least partly attributed to a favourable combination of decent transfer integrals and a low degree of dynamic disorder.

Unfortunately, it is challenging to predict the crystal structure of a newly synthesized molecule^29^. It is thus difficult to translate the insights gained from rubrene into a molecular design strategy. A strategy on the other hand that allows making the molecules more rigid in their crystal would reduce the amplitudes of intermolecular vibrations and therefore the level of dynamic disorder regardless of the exact molecular stacking in its crystal structure.

### 2,7-Dioctyl[1]benzothieno[3,2–b][1]benzothio-phene

**Figure 2:**
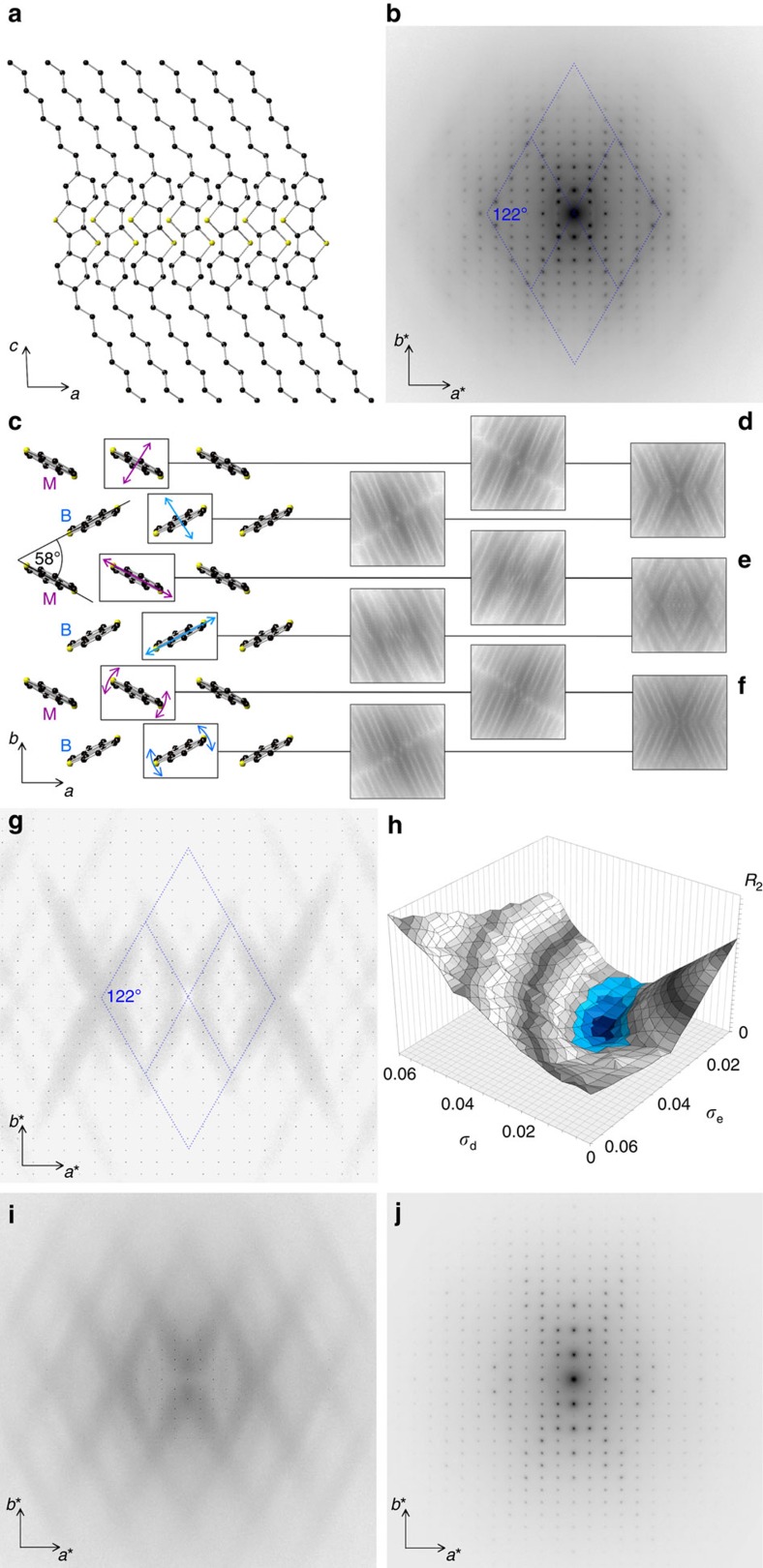
Structure of C8-BTBT and streak formation of different thermal modes with refinement. (**a**) Cross-sectional view of the C8-BTBT structure. (**b**) Experimental diffraction pattern of C8-BTBT. (**c**) Top view of the C8-BTBT structure. Hydrogens and side chains are omitted for clarity. Simulation of the diffuse features for selected vibrations, first for molecules of each orientation R and G separately, then combined (**d**–**f**). (**g**) Simulated diffraction pattern that only incorporates the vibrational modes (**d**,**e**). (**h**) Discrepancy of the simulated and experimental diffraction data expressed as the least square residual for different amplitudes for vibrations (**d**,**e**). (**i**) Simulated diffraction pattern based on MD simulation. (**j**) Experimental diffraction pattern of C8-BTBT taken at 100 K.

C8-BTBT is another promising organic semiconductor^16^. Figure 2a,c show the crystal structure of C8-BTBT, which has been published in ref. 30 and which is in good agreement with our electron diffraction results. C8-BTBT diffraction patterns are of higher quality than previously investigated organic crystals with an unprecedented low level of background noise, indicating an absence of large atomic displacements^31^. Figure 2b shows a C8-BTBT diffraction pattern presented in the same logarithmic intensity scale used in Fig. 1. Diffuse features should be visible at this intensity scale, in particular given the low level of background noise. However, similar well-defined diffuse features as in Fig. 1 are absent in C8-BTBT and only close examination reveals faint streaks.

These diffuse features form a diamond-shaped pattern with streaks running through the central (000) reflection at an angle of ∼122° and higher-order streaks in parallel. The diffraction pattern can be identified by comparison with simulations as the [001] zone-axis pattern. Figure 2b is oriented so that (h00) reflections lie horizontally and (0k0) reflections lie vertically with spatial frequencies of 0.169 and 0.127 Å^−1^, respectively. Figure 2c shows the corresponding crystal structure of C8-BTBT in identical orientation; side chains and hydrogens are omitted for clarity. The mechanism that leads to the formation of the diffuse streaks can be explained with Bragg's law and has been visualized in ref. 13. It is important to note that the direction of a diffuse streak is always perpendicular to the orientation of the thermally vibrating molecule. The unit cell of C8-BTBT contains molecules at two orientations, indicated in Fig. 2c as M (magenta) and B (blue), stacking at an angle of 58°. Molecules of each orientation create a set of diffuse streaks and the diamond-shaped pattern in C8-BTBT is, exactly as the diffraction pattern of TMTES-P and rubrene, a result of these molecular orientations, resulting in a streaking angle of 180°−58°=122° (Fig. 2b). The distance of the streaks from the central (000) reflection describes the projected, interatomic spacing in the BTBT core. For example, the first-order streak lies at 0.79 Å^−1^, which corresponds to a real space distance of 1.26 Å—the typical interatomic distance in the conjugated core when projected onto [001] direction. Different than TIPS-P, TMTES-P or rubrene whose conjugated cores purely consist of carbons, the thiophene rings in the BTBT cores introduce a larger variety of interatomic distances. This translates into a larger variety of reciprocal distances and explains the broadening of the diffuse streaks in Fig. 2b, similar to the broadening observed from other materials that comprise non-carbon atoms in their conjugated cores such as TESADT or diF-TESADT.

Different than C8-BTBT, the materials listed in Fig. 1 all possess a single strong vibration that clearly dominated the shape of the diffuse features. A simulation based on that single vibrational mode was therefore sufficient to achieve a close match between simulated and experimental diffraction patterns. The situation is different for C8-BTBT. Given the position of the side chains, there remain three intermolecular vibrations of the conjugated cores (Fig. 2c): Two translational modes, including the vibration in *π*–*π* stacking direction (Fig. 2d) and the short axis of the BTBT core (Fig. 2e), as well as a libration mode in direction of its long axis (Fig. 2f). It is shown how each of these vibrational modes creates diffuse features in reciprocal space. In Fig. 2c, the resulting diffraction pattern is first shown if only molecules of orientation M or B are considered in the simulation and then if thermal vibrations of molecules in both orientations are included. However, comparison with the experimental data of Fig. 2c shows that none of these vibrational modes alone is sufficient to achieve a good agreement, but that a combination of these modes is required. A quantitative refinement that follows the method in ref. 13 shows that atomic displacements from the libration mode (iii) must be small. The simplest approximation of the thermal vibrations is thus given by a model that incorporates both translational modes. The resulting diffraction pattern is shown in Fig. 2g. The characteristics of the experimental pattern are well represented, despite the strong simplification of the vibrational picture. Smaller amplitude thermal modes that were omitted in the simulation only have a perceptible effect at higher spatial frequencies, that is, at the edges of the experimental diffraction pattern. This explains the drop-off in intensity in the experimental data that is not reproduced in the simulation. Nevertheless, the simulation reproduces all diffuse features in the experimental diffraction pattern, which is a good indication that the dominant, large-amplitude vibrational modes are well described. If the amplitude of these modes is varied and refined to match the experimental data, a plot shown in Fig. 2h emerges. The discrepancy between simulated and experimental data is minimal if amplitudes for the molecular displacements are chosen of *σ*_d_≈*σ*_e_≈0.024 Å. This figure is four to five times smaller than the typical values observed for the molecules displayed in Fig. 1, indicating that C8-BTBT might be significantly less affected by thermal vibrations than previously investigated molecules. The circular shape of the Gaussian suggests isotropic displacements of the BTBT cores around their equilibrium position. This is plausible as in comparison with the molecules of the TIPS-P family (Fig. 1), the conjugated cores of C8-BTBT are uniformly surrounded by its in-plane neighbours. Details about the simulation are described in Supplementary Note 1; C8-BTBT film thickness and amplitude refinement are shown in Supplementary Fig. 8. The simulation is available online^32^, including detailed explanations and sample files for TIPS-P, diF-TESADT and C8-BTBT.

Figure 2i shows the same C8-BTBT electron diffraction pattern calculated based on the result of molecular dynamics (MD) simulations. Following the method given in ref. 6, the interatomic distances in the BTBT core have been adjusted to match the experimental structure. The diffraction pattern computed from MD simulations shows a more realistic description of the diffuse noise background in comparison with the experimental data. This is a result of the additional thermal vibrations that are naturally included in the MD simulation in comparison with the simplified model that only accounts for the two dominant thermal vibrations. Abandoning the assumption of rigid side chains that guaranteed a stringent spatial periodicity in the simplified vibrational model, the MD generated diffraction pattern shows a more realistic intensity drop-off for higher spatial frequencies. The diffuse features that stem from intermolecular thermal vibrations of the BTBT cores are reproduced correctly by the MD simulation. Comparisons between MD-calculated diffraction patterns and the experimental data can also be used to improve these calculations. For example, MD simulations of C8-BTBT have been found to slightly overestimate the amount of thermal disorder in the crystal, which is in agreement with similar results that have been previously found for TIPS-P^13^. One possibility to improve this is by constraining the bond lengths in the MD simulation at their equilibrium distance. Such constraints mimic the quantum nature of the high-energy stretching vibrations, which are unable to gain thermal energy at the temperatures of interest, that is, they do not contribute to the disorder.

Figure 2j shows the same diffraction pattern than Fig. 2b but taken at a temperature of 100 K. Diffuse features including the noise background are visibly reduced, supporting the picture that the diffuse intensity is a fingerprint of large thermal atomic displacements. Low-temperature diffraction pattern of other materials such as diF-TESADT (Supplementary Fig. 13) or TIPS-P (ref. 13) also show a strong reduction in their diffuse features but remain more visible than C8-BTBT patterns, which further corroborates our finding that the low-frequency, large-amplitude vibrations present in these materials are absent in C8-BTBT.

### Diffraction analysis

**Figure 3:**
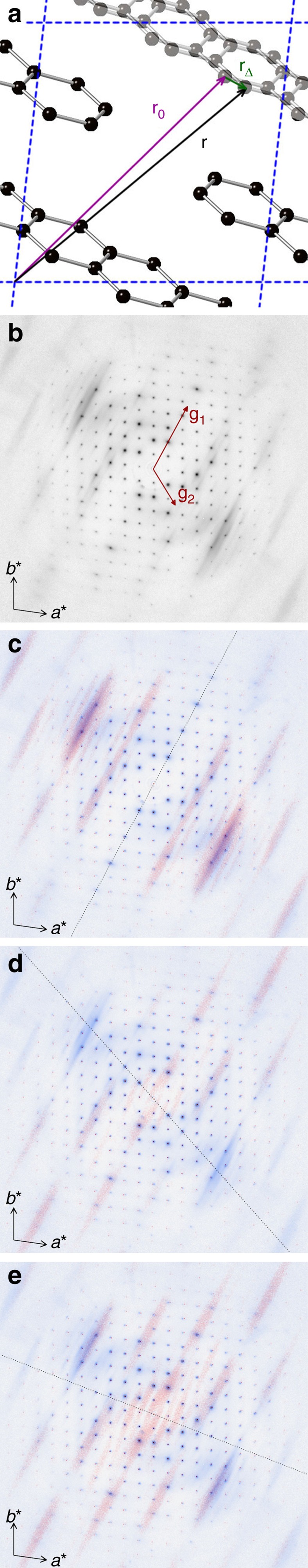
Analysis and simulation of TIPS-P diffraction pattern. (**a**) Structure of TIPS-P with real space vector **r**=**r**_0_+**r**_Δ_ describing the position **r** of a single carbon atom in its unit cell as superposition of its equilibrium position **r**_0_ and thermal displacement **r**_Δ_. (**b**) Experimental diffraction pattern of TIPS-P from Fig. 1a, corrected for its noise background to better contrast the streak intensity. The missing (000) reflection is an artefact of the background correction due to the high intensity drop-off close to the centre of the diffraction pattern. (**c**–**e**) Simulated diffraction patterns with the conjugated pentacene cores displaced parallel to its long axis (**c**), along its short axis (**d**), in *π*–*π* stacking direction (**e**). The simulated diffraction patterns (red) are laid over the experimental diffraction patterns (blue) for easier comparison. The dotted line indicates the direction perpendicular to the modelled molecular displacement **r**_Δ_.

An in-depth analysis of the diffraction patterns helps to explain the discrepancy of the thermal amplitudes in C8-BTBT and the materials presented in Fig. 1. The structure factor *S∼exp(-i***g*****·*****r**) describes the scattering potential of an atom at position **r** in the unit cell of the crystal (Fig. 3a) into a reciprocal space vector **g** in the diffraction pattern (Fig. 3b)^33^. If the atomic position is written as **r***=***r**_0_*+***r**_Δ_, whereas **r**_0_ describes the atom's equilibrium position and **r**_Δ_ its thermal displacement (Fig. 3a), the structure factor splits in two terms exp(−*i***g*****·***(**r**_0_*+***r**_Δ_))=exp(−*i***g*****·*****r**_0_) *exp(−*i***g*****·*****r**_Δ_). The first expression describes the perfect crystal structure where every atom sits exactly at its equilibrium position **r**_0_, that is, without thermal vibrations. If it was only for this term, the diffraction pattern would represent the perfect periodicity of the crystal structure with all diffraction intensity being scattered in the Bragg reflections. However, the second exponential term that describes thermal atomic displacements **r**_Δ_ disturbs this periodicity and as a result electrons are scattered in the reciprocal space between the Bragg reflections. It is noteworthy that **g**·**r**_Δ_ is zero for reciprocal space vectors **g** with **g**⊥**r**_Δ_. Therefore, a thermal vibration creates no diffuse streak intensity in parts of the diffraction pattern, which are described by a reciprocal space vector **g** that is approximately perpendicular to the direction of the thermal displacement **r**_Δ_. Figure 3b shows the same experimental diffraction pattern of TIPS-P as in Fig. 1b but corrected for its noise background, which helps to better contrast the well-defined diffuse features. Two arbitrary reciprocal space vectors **g**_**1**_ and **g**_**2**_ are drawn. It is noteworthy how no diffuse features are observed in the experimental diffraction pattern along points of the reciprocal space described by vectors **g** parallel or almost parallel to **g**_**1**_. Figure 3c–e show simulations of the Fig. 3b diffraction pattern based on different translational modes, with the conjugated core vibrating in direction of its long axis (Fig. 3c), in the direction of its side chains (Fig. 3d) and in the direction of the *π*–*π* staking (Fig. 3e). Each simulated diffraction pattern is coloured red for easier comparison and laid on top of the experimental pattern shown in Fig. 3b (coloured blue). The dotted lines in Fig. 3c–e show the direction perpendicular to the direction of the respective molecular displacement **r**_Δ_. It is worth noting that diffuse features are strongly reduced or even absent in the simulated diffraction patterns (red) along these directions as expected.

From the simulations (Fig. 3c–e) we know that if the conjugated cores would be noticeably displaced in any other direction than the core's long axis, we would observe diffuse intensity (red) in the direction of **g**_**1**_. Therefore, the absence of diffuse intensity along **g**_**1**_ signals that large atomic displacements arise only in long-axis direction, as substantial atomic displacements in any other direction would cause such diffuse features in the experimental diffraction data. The same analysis can be applied to all materials shown in Fig. 1. We chose TIPS-P, as the sulfur and fluorine atoms in TESADT and diF-TESADT create a larger variety of interatomic distances, resulting in broader diffuse streaks that makes them slightly harder to visualize.

To summarize, the comparison between simulated and experimental data reveals not only that amplitudes in direction of the conjugated core's long axis are largest but also that displacements caused by other thermal vibrations are comparatively small. In other words, the conjugated cores of the materials shown in Fig. 1 experience sufficiently large force constants, to reduce thermal amplitudes in all directions but along their long axes.

## Discussion

Using molecular mechanics simulations we estimated the effective force constants keeping the conjugated cores of TIPS-P and diF-TESADT in equilibrium (Supplementary Note 2). The force constant is the smallest, that is, the potential energy surface is the shallowest, in the direction of the long axis of the conjugated core. In contrast, the force constants for displacements side chain and *π*–*π* stacking direction are at least twice as high in both molecules (Supplementary Fig. 14). The simulations for these materials are therefore in good agreement with our diffraction experiments and support the result that vibrations of the conjugated cores exhibit the largest amplitudes in long-axis direction. Along this direction, only few intermolecular interactions keep the core in place and it is not surprising the resulting motion is the largest.

A much larger force applies in the direction of the side chains, that is, in out-of-plane direction. Deviation from equilibrium is only possible via deformation of very rigid C–C bonds or if the side chain follows the vibration of the conjugated core. The first scenario would directly result in a large intramolecular force constant and the latter strongly increase intermolecular van der Waals forces, as adjacent side chains are greatly enmeshed. Force-field analysis shows that if the side chains followed the out-of-plane vibration of the conjugated core, the increase in van der Waals forces would be equally large than for a vibration in *π*–*π* stacking direction but still energetically favourable compared with a deformation of the C–C bonds (Supplementary Tables 1 and 2). This explains the experimental data, suggesting that there is no correlated motion of the conjugated core and the side chain as it has been assumed in ref. 13 and shown for TIPS-P, diF-TESADT and C8-BTBT in Supplementary Fig. 1.

In contrast, the contribution of the side chains to the force constant of in-plane vibrations is marginal (Supplementary Note 3), that is, it is essential to consider van der Waals and *π*–*π* interactions to explain the large force constant in direction of the *π*–*π* stacking. Although only few interactions contribute to the force constant in the direction of the long axis, the brick-wall stacking motif produces a large number of weak interactions between adjacent molecules in *π*–*π* stacking direction that accumulate to a considerable force constant. The missing evidence in experimental diffraction pattern of large atomic displacements along this direction (Fig. 3) tells us that these *π*–*π* stacking interactions are sufficient to stabilize the conjugated core.

This is an important result, as it means that not only the attachment of side chains but also the accumulation of individually weak force constants in *π*–*π* stacking direction are sufficient to reduce the amplitude of thermal vibrations. Therefore, systems in which the conjugated cores are encapsulated in all three dimensions by one of these mechanisms should provide the optimum molecular packing to achieve minimum vibrational amplitude and dynamic disorder.

As the number of interactions stabilizing the conjugated core along its long axis is small, such optimum situation can only be realized if the side chains are attached in this direction, as it is the case for C8-BTBT (Fig. 2a). In this molecular design, long-axis vibrations are suppressed in amplitude by the side chains and vibrations perpendicular to the long axis are suppressed by a large number of weak interactions, adding up to a considerable force constant. In C8-BTBT the S–S interaction might form an important contribution to this force constant, as they have been proposed in literature to reduce the *π*–*π* stacking distance^34^.

We believe that the high mobilities reported for C8-BTBT are possible because of the molecular design of the molecule: The reduced amplitude of thermal vibrations creates less dynamic disorder, allowing for more extended wave function delocalization. Such stiffening of vibrational frequencies is therefore expected to benefit the charge carrier mobility^10^.

**Figure 4:**
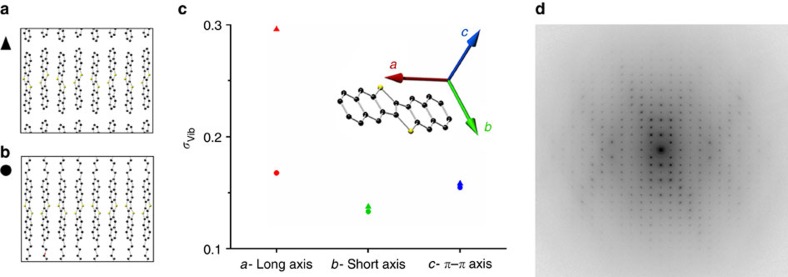
Molecular vibrations in materials with a similar molecular design than C8-BTBT. Cross-sectional view of (**a**) DNTT and (**b**) C10-DNTT. (**c**) Thermal amplitudes as computed from MD simulations along different axis *a*, *b* and *c* for DNTT (triangles) and C10-DNTT (circles). (**d**) Experimental diffraction pattern of C10-DNTT at room temperature, showing only very weak diffuse characteristics similar to those in C8-BTBT.

Fortunately, these results are not restricted to C8-BTBT but equally apply to other high-mobility molecular semiconductors adopting a similar molecular design. We investigated C10-DNTT. C10-DNTT stacks in the same manner as C8-BTBT, that is, it is encapsulated in out-of-plane direction by its alkyl side chains and in-plane by parallel aligned *π*-stacks of adjacent molecules as shown in Fig. 4b. A room temperature diffraction pattern of C10-DNTT (Fig. 4d) exhibits weak diffuse features analogous to those in C8-BTBT. The similar characteristic of the diffuse streaking is not surprising, as the molecular and crystal structures are similar, creating energetic potentials that allow similar thermal vibrations. Although we have not yet performed a quantitative refinement for C10-DNTT, the diffuse streaks appear equally weak or even weaker than in C8-BTBT. This makes sense in respect of the longer molecular DNTT core that comprises two additional benzene rings. The accumulated forces between adjacent molecules are thus likely to be even larger than in C8-BTBT, which further reduces the amplitude of thermal vibrations.

DNTT is an interesting structure as the unalkylated DNTT parent-conjugated core (Fig. 4a) stacks in a very similar crystal structure than the alkylated C10-DNTT, allowing us to investigate the stabilizing effect of the side chains in more detail. Even though we found that MD simulations overestimate the vibration amplitudes, a relative comparison between the materials is still insightful. Figure 4c shows the amplitude as predicted by MD simulations along all three molecular axis in unalkylated DNTT (triangles) and C10-DNTT (circles). As expected, the attachment of the side chains stabilizes the conjugated core in all directions. However, the strongest stabilization clearly occurs in the long-axis direction where vibrations of the non-alkylated molecule are strongest, exactly as we found for the materials of Fig. 1 that experience no stabilization through their side chains for this vibration. This result is in excellent agreement with the recent observation that charge carrier mobility in C_*n*_-DNTT increases with increasing side chains length^35^.

The MD simulations of C10-DNTT and DNTT supports our assumption that conjugated cores with their side chains attached to their long axis are better encapsulated. Such simulations are important as vibrations parallel to the incident electrons, that is, in long-axis direction for C8-BTBT, create hardly any signal in electron diffraction patterns. We have tried to overcome this shortage of the experimental TEM study by investigating two BTBT derivatives that have been synthesized recently with shorter, bulkier side chains^36^. These side chains tend to cause an inclination of the molecular cores respective to the substrate, which allows us to experimentally investigate the long-axis vibration in these molecules (Supplementary Note 4). We have chosen the BTBT derivatives 2,7-di-tertbutyl[1]benzothieno[3,2–b][1]benzothiophene and bis(trimethylsilyl)[1]benzothieno[3,2–b][1]benzothiophene, owing to the large angles of 40.5° and 51.3° between their conjugated cores and the substrate normal (Supplementary Figs 15 and 16). The resulting orientation of the conjugated cores enables us to compare the strength of intermolecular vibrations along all three directions of the conjugated core, similar to the analysis of TIPS-P in Fig. 3. This shows that the long-axis vibration is not dominant in both molecules, which is qualitatively different to the large-amplitude, one-dimensional vibrations found in the molecules presented in Fig. 1, and further corroborates our assumptions that our results generally apply to molecules that have their side chains attached along their long-axis direction.

We emphasize that sufficiently large transfer integrals are still required for obtaining high mobilities and therefore not every molecule with its side chains attached to its long axis will be a good semiconductor. However, our data suggest that these molecules do not rely to the same extent on the rare combination of good transfer integrals and small electron–phonon coupling as it is found in rubrene, to be a high-performance semiconductor. Instead, large-amplitude vibrations are intrinsically suppressed by the molecular structure, meaning that the transfer integrals in these materials tend to be less compromised by dynamic disorder.

To summarize, we observe that large-amplitude thermal vibrations that are most harmful for charge transport are commonly found in molecules with their side chains attached to the short axis of their conjugated core. This applies to brick-wall stacked molecules such as TIPS-P and diF-TESADT, and also to molecules such as TMTES-P and rubrene, which crystalize in a *π*–*π* herringbone packing motif. Unless the sensitivity of the transfer integral to these vibrations is not exceptionally low as in rubrene, the resulting energetic disorder will compromise performance in these molecules. Interestingly, analysing TEM diffraction data shows that large amplitudes are absent in the direction of their side chains and in *π*–*π* stacking direction. MD simulations confirm that molecules experience a large force constant in direction of their side chains. Equally large force constants are found in *π*–*π* stacking direction due to the large number of aggregated van der Waals and *π*–*π* stacking interactions, and indeed experimental diffraction data confirms that large-amplitude vibrations are suppressed in this direction. However, in long-axis direction the number of weak interactions between molecules is too small to produce sufficiently large force constants that would reduce the amplitudes of thermal vibrations. These insights provide a general molecular design strategy for reducing the amplitudes of thermal vibrations in molecular semiconductors. By substituting the side chains along the long axis of the molecule, large amplitudes in that direction can be suppressed, while the conjugated cores are stabilized by large aggregated force constants in other directions. We have shown that two representatives of this design rational—C8-BTBT and C10-DNTT—exhibit intermolecular thermal vibrations with significantly lower amplitudes compared with molecules of the TIPS-P family. This suggests that the associated low degree of dynamic disorder might be an important factor, contributing to the promising charge carrier mobilities that have been reported for these materials. We hope that the molecular design strategy presented in this study will help to achieve similarly high carrier mobilities in a wider range of organic semiconductors.

## Methods

### TEM sample preparation

An 18.53 wt% polystyrene sulfonic acid solution in water from Scientific Polymer Products was spin coated onto a cleaned glass substrate using a Laurell Technologies WS-400B-6NPP spin coater operating at 2,500 r.p.m. for 30 s. The organic semiconductor was dissolved as a 0.01 wt% solution in tetralin and drop-cast onto the substrate, which was constantly held at 60 °C in a nitrogen atmosphere that has been almost fully saturated with tetralin. After slow evaporation of the tetralin solvent, the sample was removed from the glovebox and a few droplets of deionized water were applied to dilute the polystyrene sulfonic acid layer. After a few minutes, organic crystals floated on the water surface where they were easily picked up by a TEM grid.

### Experimental TEM work

All experimental TEM work has been conducted on a Philips CM30 microscope that runs with a LaB6 source at 300 kV. We operated the microscope at low-dose settings to prevent sample degradation and recorded all diffraction data on high dynamic range imaging plates from Ditabis, which enabled us to use the microscope without beam stop and allowed us to obtain sufficient pixel depth for quantitative analysis despite low-dose settings.

### Data Availability

Additional data related to this publication is available at the University of Cambridge data repository (https://www.repository.cam.ac.uk/handle/1810/253572).

## Additional information

**How to cite this article:** Illig, S. *et al.* Reducing dynamic disorder in small-molecule organic semiconductors by suppressing large-amplitude thermal motions. *Nat. Commun.* 7:10736 doi: 10.1038/ncomms10736 (2016).

## Supplementary Material

Supplementary Information Supplementary Figures 1-16, Supplementary Tables 1-2, Supplementary Notes 1-4, Supplementary Methods and Supplementary References
