# Supplementary material for: Reducing dynamic disorder in small-molecule organic semiconductors by suppressing large-amplitude thermal motions
Source: Nat Commun. 2016 Feb 22;7:10736. doi: 10.1038/ncomms10736 (PMC4764867; doi:10.1038/ncomms10736)
Supplement: Supplementary Information — Supplementary Figures 1-16, Supplementary Tables 1-2, Supplementary Notes 1-4, Supplementary Methods and Supplementary References [file ncomms10736-s1.pdf]

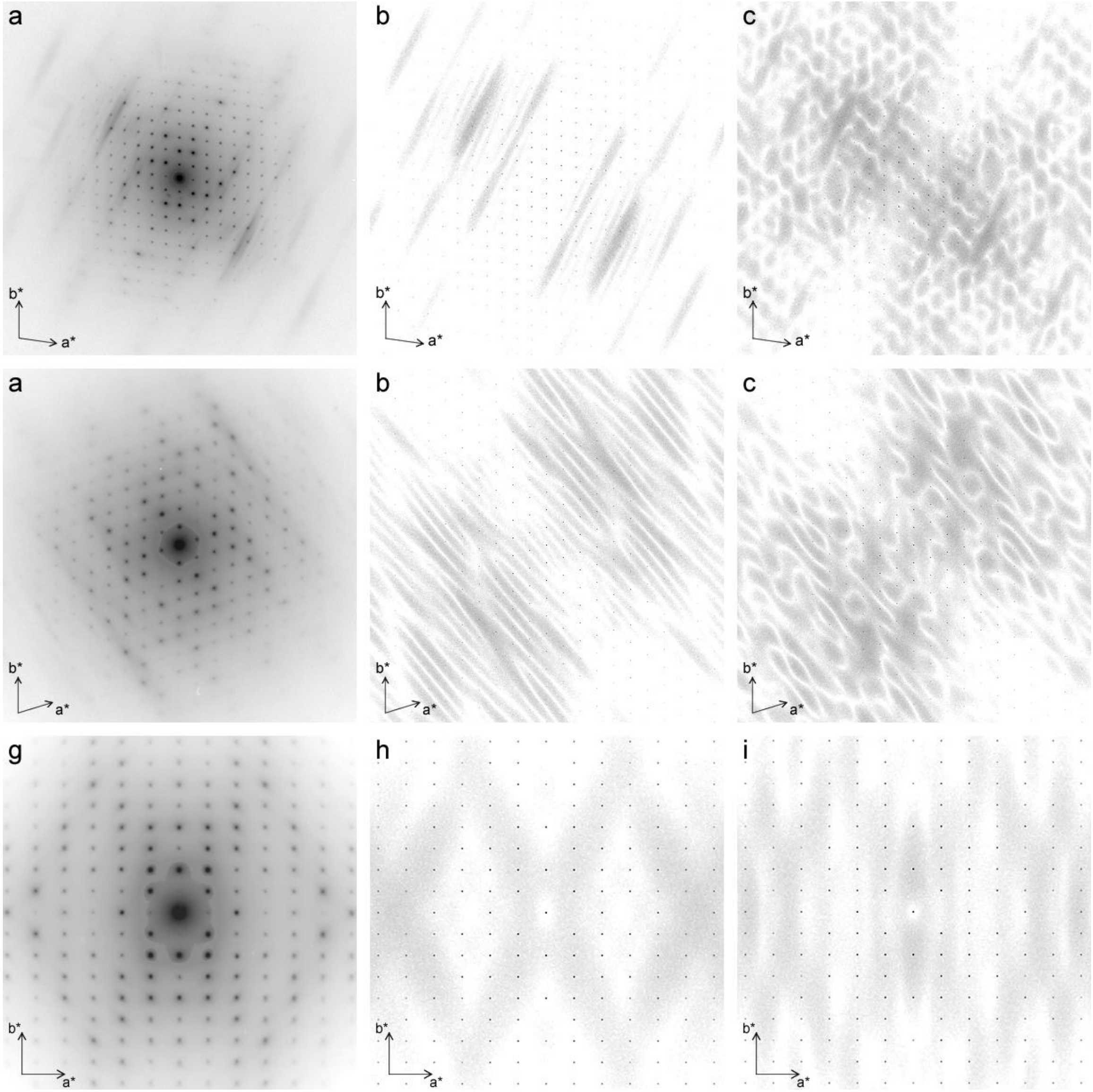


**Supplementary Figure 1: TEM diffraction data and simulations suggesting that side chains do not follow the vibrations of the conjugated core.** a-c, TIPS-P. d-f, DiF-TESADT. g-i, C8-BTBT. a, d, g Experimental pattern. b, e, h Simulated diffraction pattern without the side chains following the displacement of the conjugated core. c, f, i Simulated diffraction pattern with the side chains following the displacement of the conjugated core. All diffraction patterns show the [001] zone axis.

(1) Supplementary information

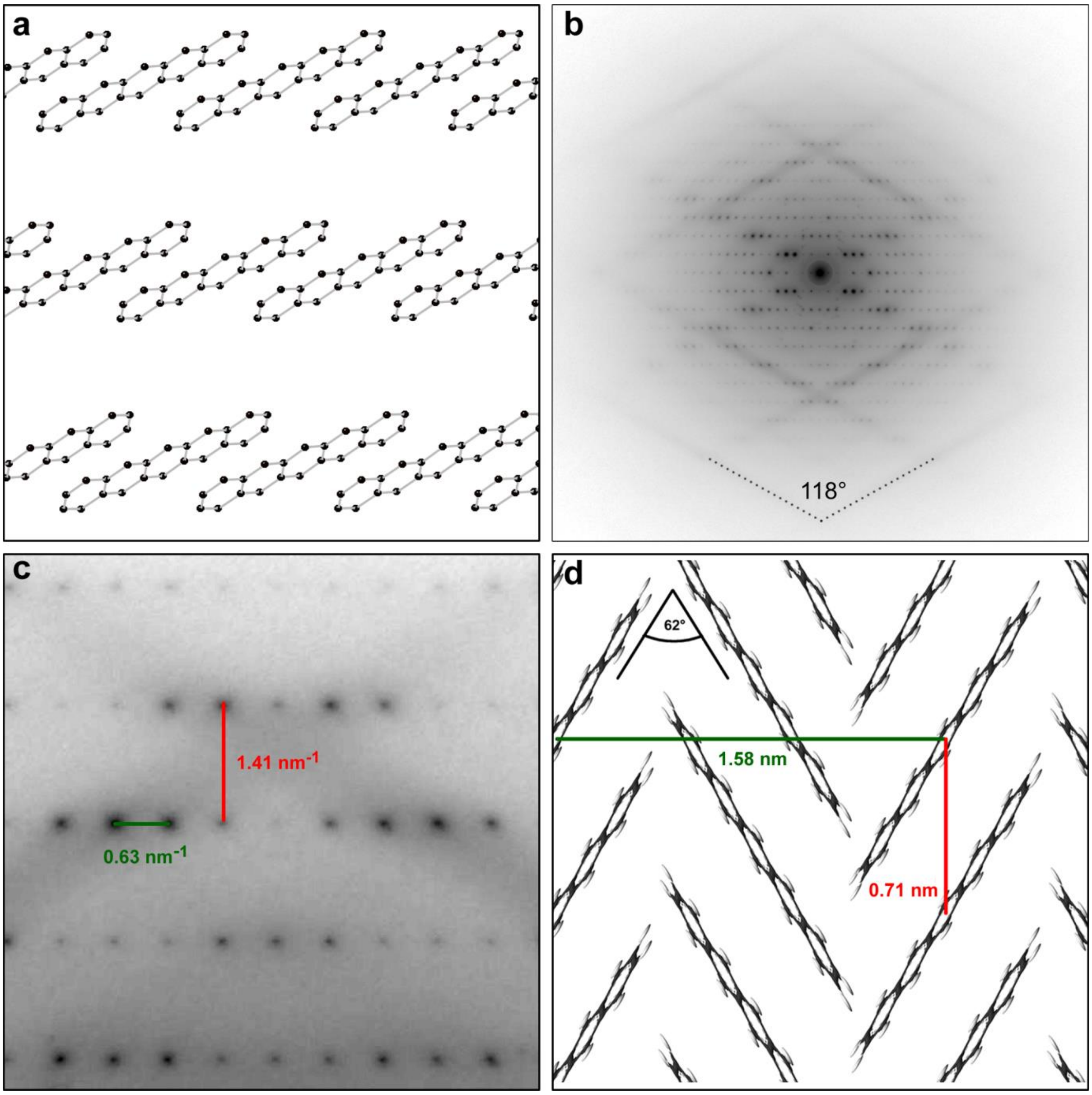


**Supplementary Figure 2: Thin-film structure of 1,4,8,11-tetramethyl-6,13-triethylsilylethynylpentacene (TMTES-P) from TEM diffraction pattern analysis.** a, Brick-wall stacked TMTES-P structure published in ref. [1] oriented in the [001] direction. Hydrogens and side chains are omitted for clarity. b, Electron diffraction pattern of a TMTES-P thin-film; indicating a herringbone packing motif. c, Magnification of the pattern in b with reciprocal space distances between reflections; showing that the unit cell of the thin-film structure must be larger than suggested by the structure in a. d, Reconstruction of the thin-film structure based on electron diffraction data. The structure coincides with a polymorph that has been found in powders of TMTES-P and that allowed for an in-cell refinement. The structure has been made available via the Cambridge Structural Database [2].

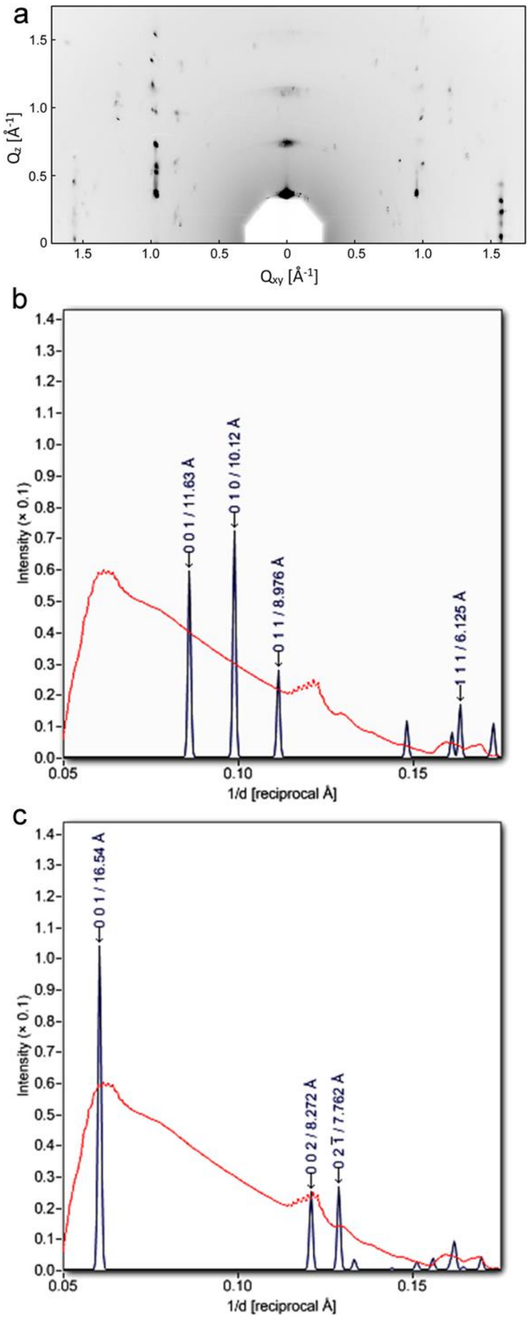


**Supplementary Figure 3: Grazing incident X-ray data confirming the TEM diffraction results showing that thin-films of TMTES-P stack in a herringbone structure.** a, Uncorrected grazing incident X-ray data of a TMTES-P thin-film. b, Simulated XRD profile based on the crystal structure published in ref. [1] and c, simulated XRD profile based on the structure published in ref. [2]. The radially integrated profile of the experimental data in (a) is shown in red. Analysis of the TMTES-P diffraction data shows that the thin films stack in the herringbone packing motif.

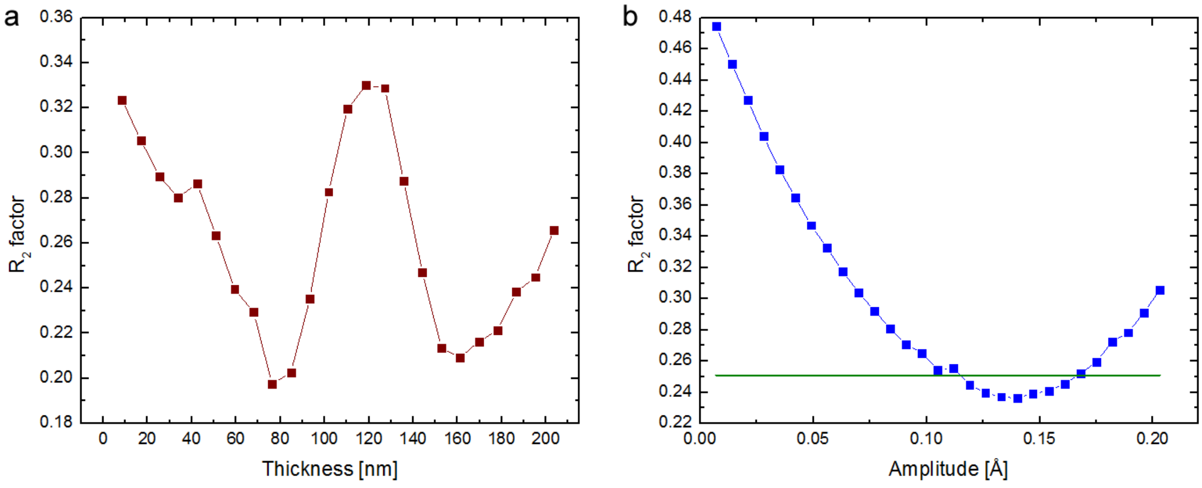


**Supplementary Figure 4: Quantitative refinement of TESADT.** a, Refinement of the film thickness. b, Refinement of the vibration amplitude with refinement error (green).

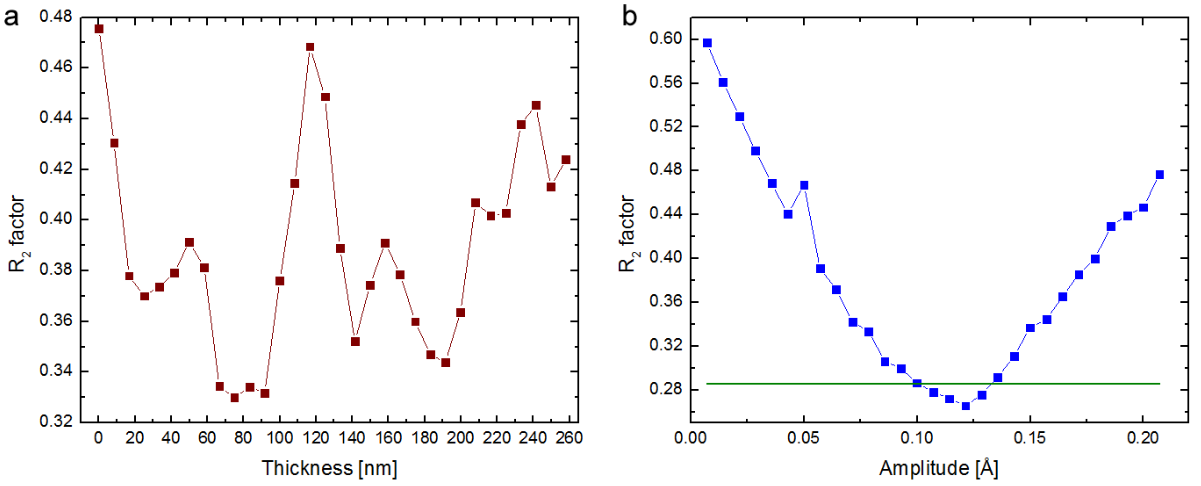


**Supplementary Figure 5: Quantitative refinement of diF-TESADT.** a, Refinement of the film thickness. b, Refinement of the vibration amplitude with refinement error (green).

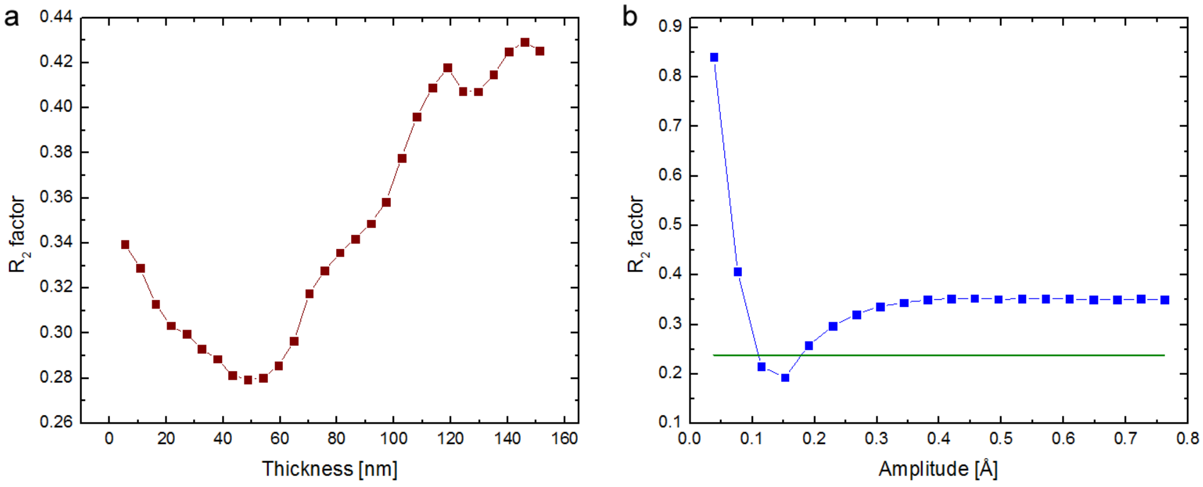


**Supplementary Figure 6: Quantitative refinement of TMTES-P.** a, Refinement of the film thickness. b, Refinement of the vibration amplitude with refinement error (green).

(4) Supplementary information

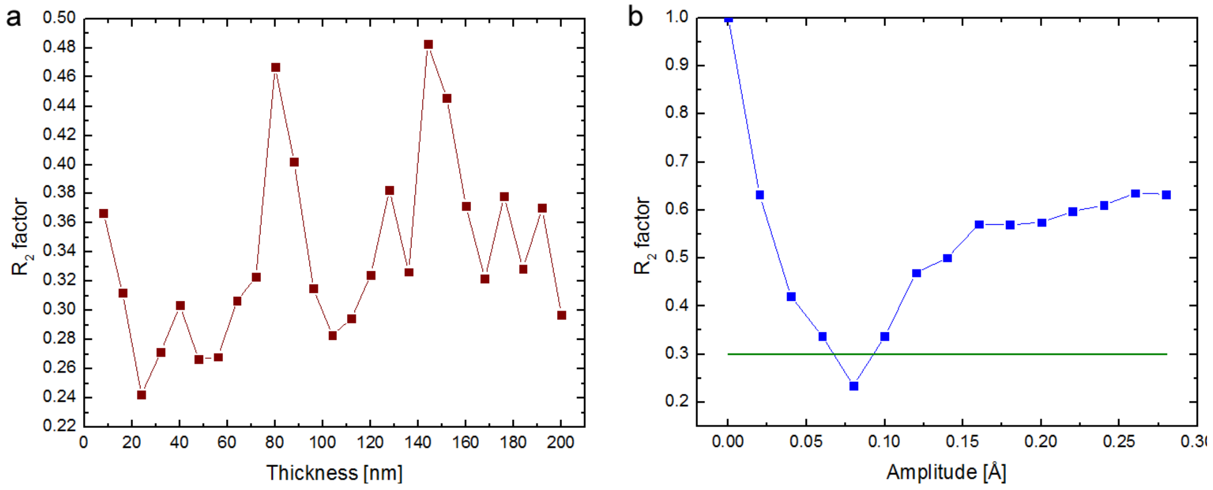


**Supplementary Figure 7: Quantitative refinement of rubrene.** a, Refinement of the film thickness. b, Refinement of the vibration amplitude with refinement error (green).

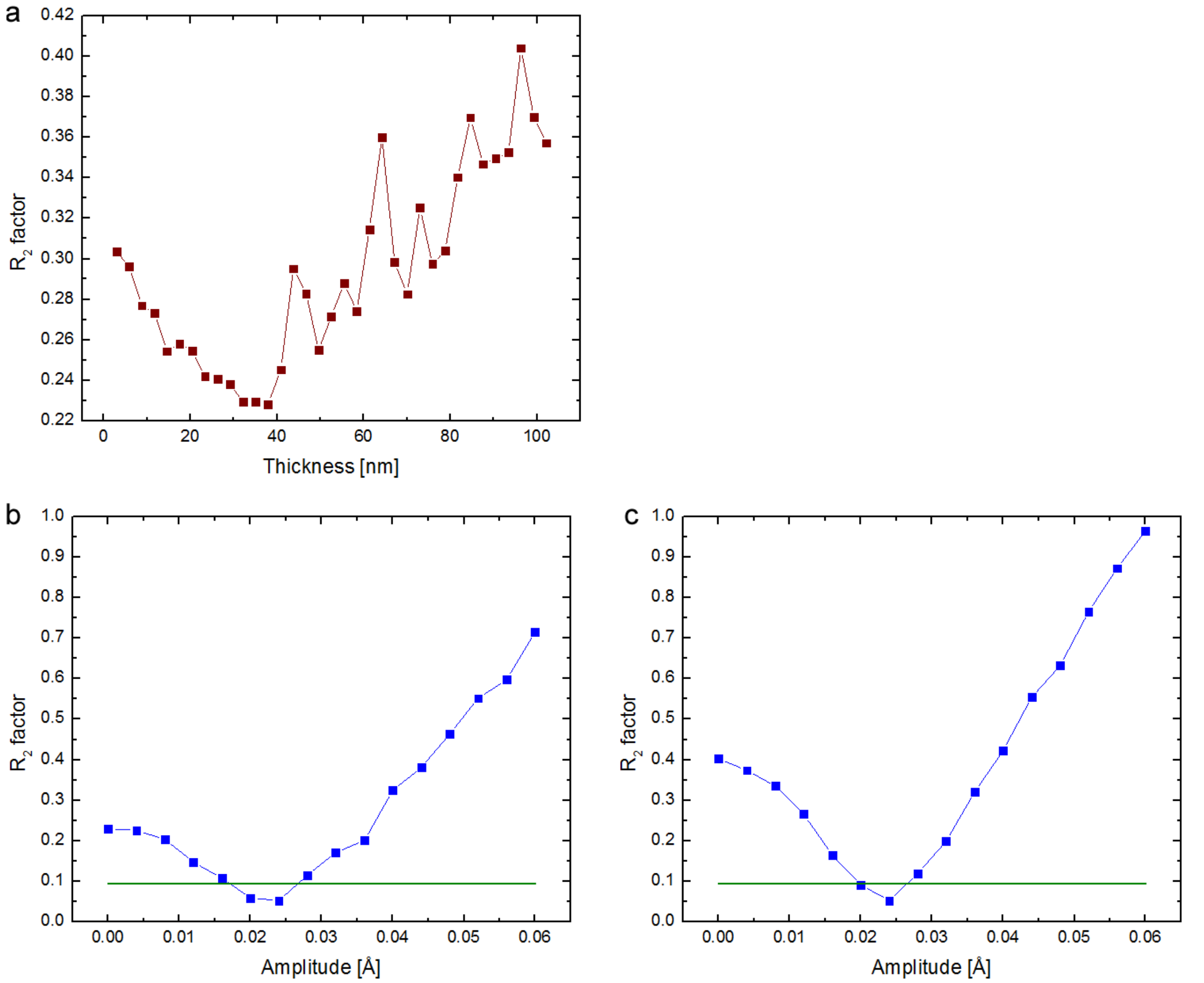


**Supplementary Figure 8: Quantitative refinement of C8-BTBT.** a, Refinement of the film thickness. b, Refinement of the vibration amplitude in direction of the short axis. c, Refinement of the vibration amplitude in direction of the π-π stacking. The refinement error is indicated in green.

(5) Supplementary information

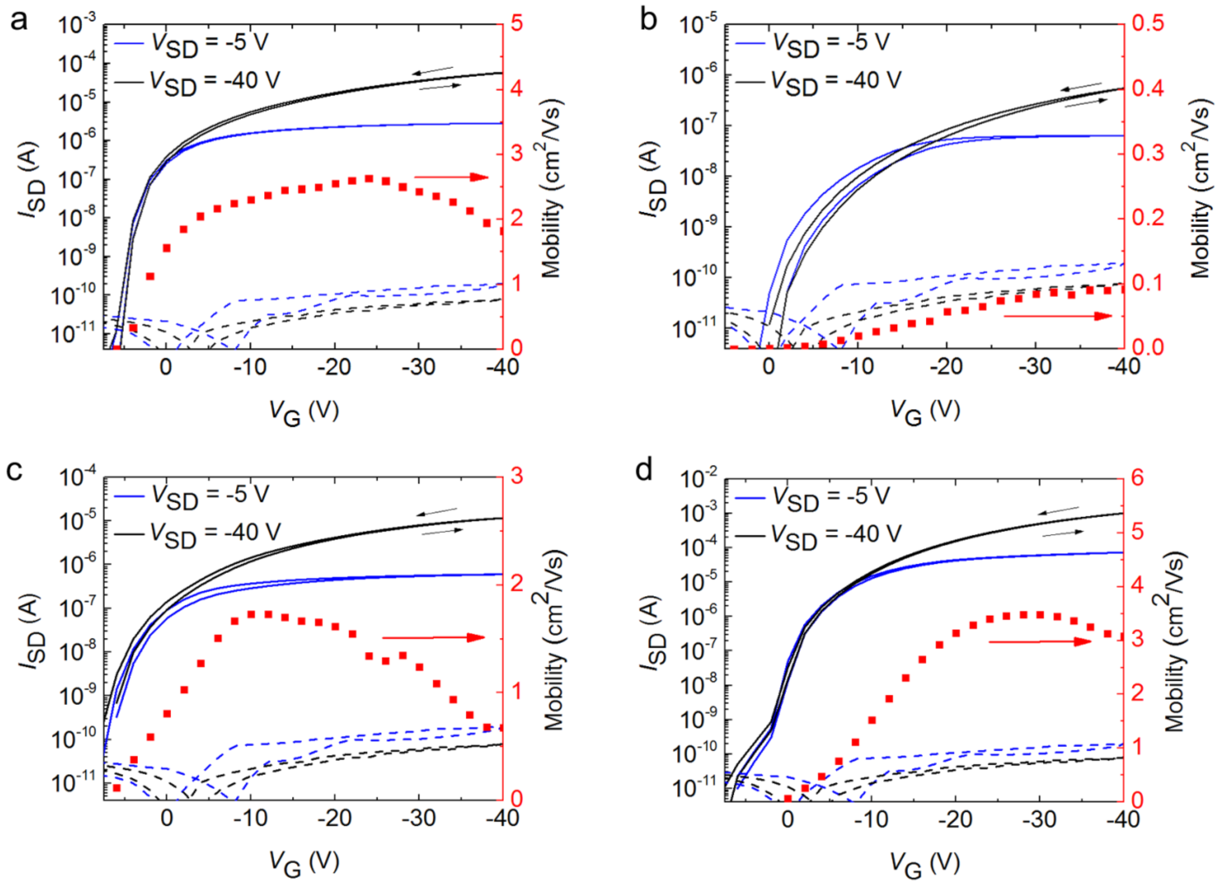


**Supplementary Figure 9:** Transfer characteristics of (a) TMTES-P, (b) TIPS-P, (c) TESADT and (d) diF-TESADT.

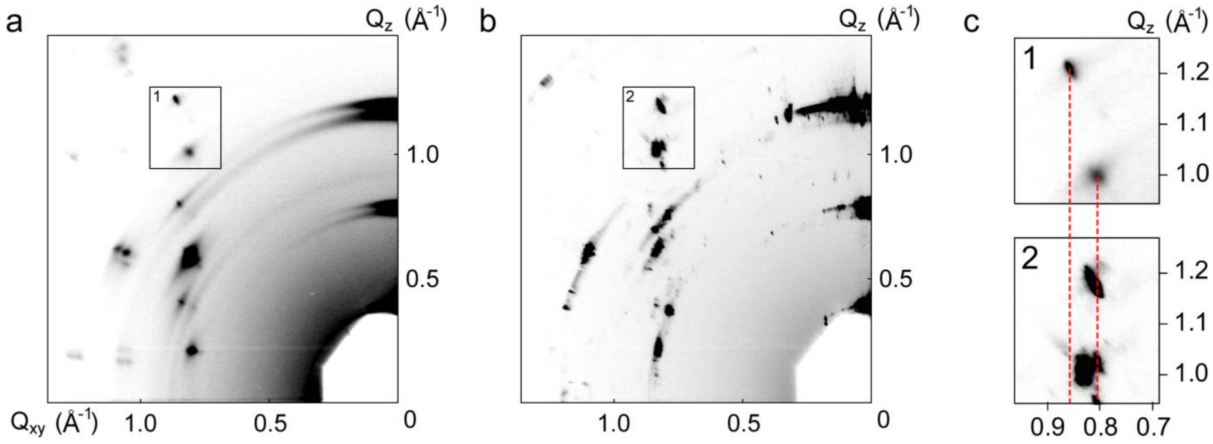


**Supplementary Figure 10:** Grazing incident diffraction patterns of spin-coated (a) and drop-casted (b) films of TIPS-P. c, Shift of the (103) and (012) reflections indicating a structural change for different processing conditions as reported in ref. 3. The data have not been corrected for curvature of the Ewald sphere, but clearly show differences in the polymorphic structure of the thin spin-coated films and the single crystals. Mobilities in the spin-coated films are of an order of magnitude higher compared to drop-casted films; probably the result of a more favourable stacking that results in increased electronic coupling.

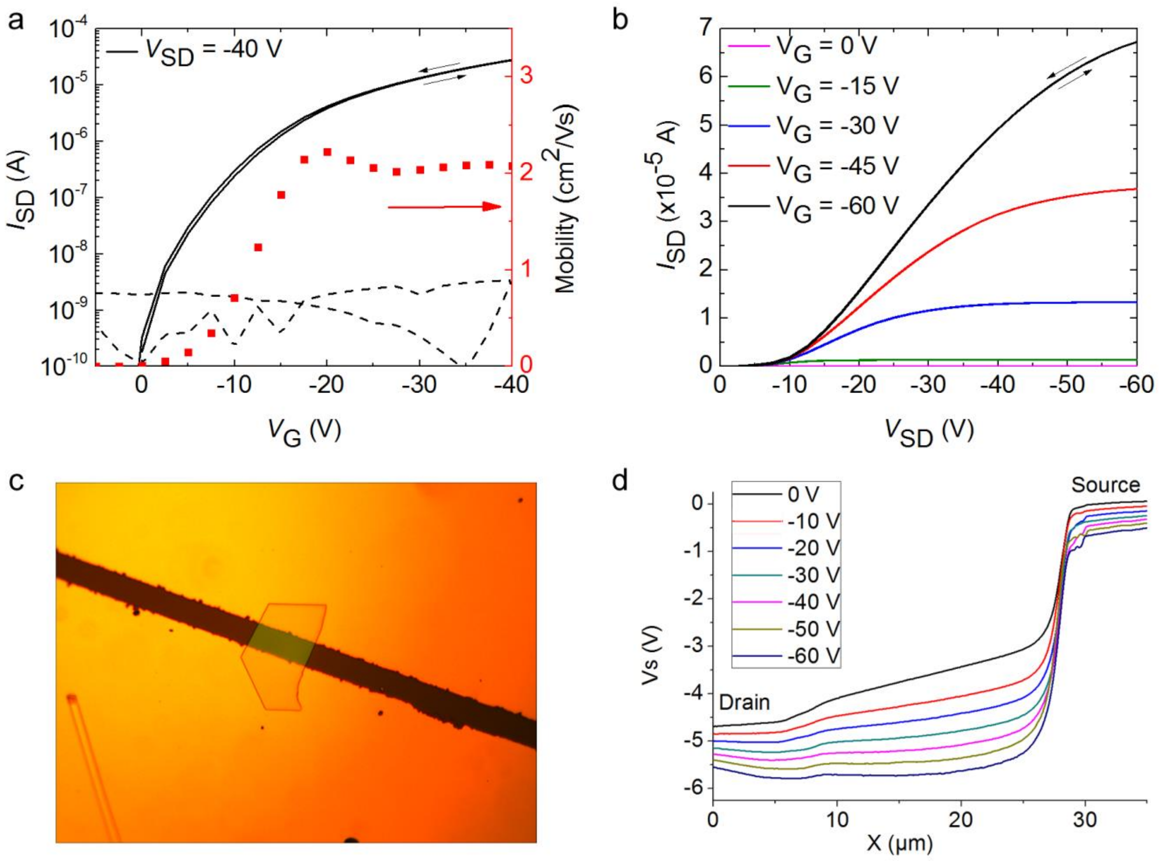


**Supplementary Figure 11:** Rubrene transfer (a) and output (b) characteristics of the bottom gate device shown in (c). Shown is the vapor grown single crystal that has been transferred on the gold source-drain electrodes. (d) Scanning Kelvin Probe measurements of the same device for different gate voltages show a major potential drop at one of the electrodes. Correcting the mobility for the contact resistance resulted in values around 15 cm$^2$/Vs as presented in Fig. 1 in the main text.

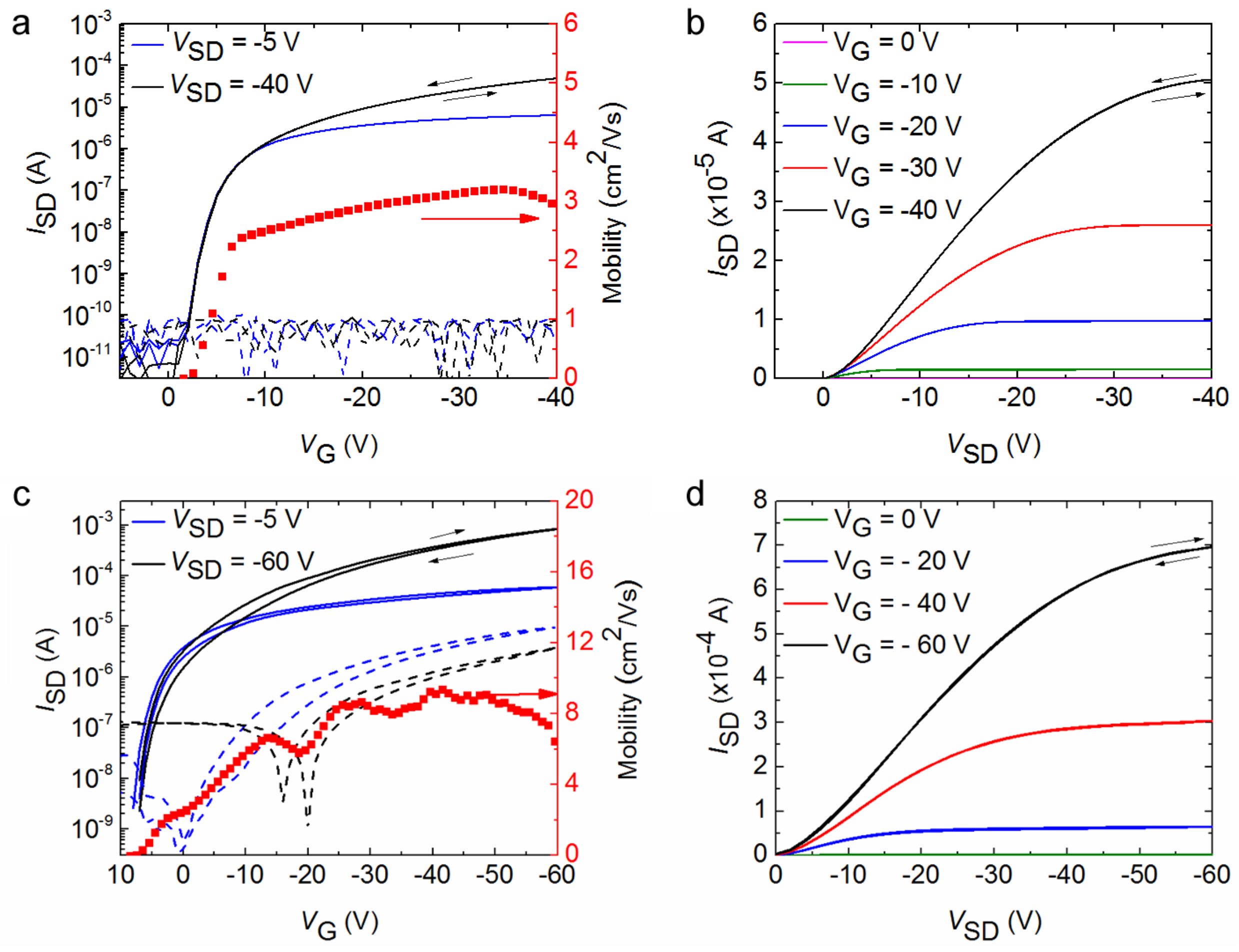


**Supplementary Figure 12:** Transfer (a, c) and output (b, d) characteristics for an evaporated, polycrystalline film of C8-BTBT (a, b) and a wedge-casted crystal of C10-DNTT (c, d) .

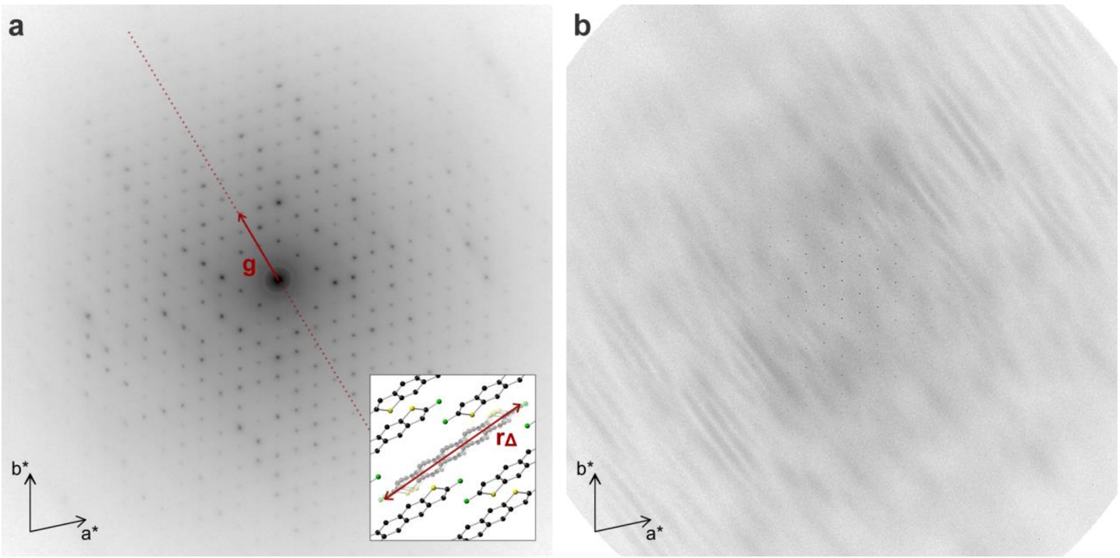


**Supplementary Figure 13: Experimental low temperature diffraction pattern of diF-TESADT and simulated diffraction pattern based on molecular dynamics (MD) trajectories** a, Diffraction pattern of diF-TESADT taken at 100 K and oriented in [001] direction. The thermal diffuse intensity is notably reduced at this temperature which validates the thermal character of these diffuse features. The inset shows the corresponding molecular structure in the same orientation. The red dotted line shows the direction of reciprocal space vectors **g** along which no diffuse features are observed. Following the argumentation in the main text, their absence indicates that large atomic displacements occur only in long-axis direction. b, Simulation of the pattern shown in (a) based on MD simulation at 100 K. Both, the diffuse streaks and the noise background of the experimental data are reproduced well. In contrast, experimental diffraction pattern of C8-BTBT (Fig. 2j in the main text) show hardly any diffuse intensity at low temperatures.

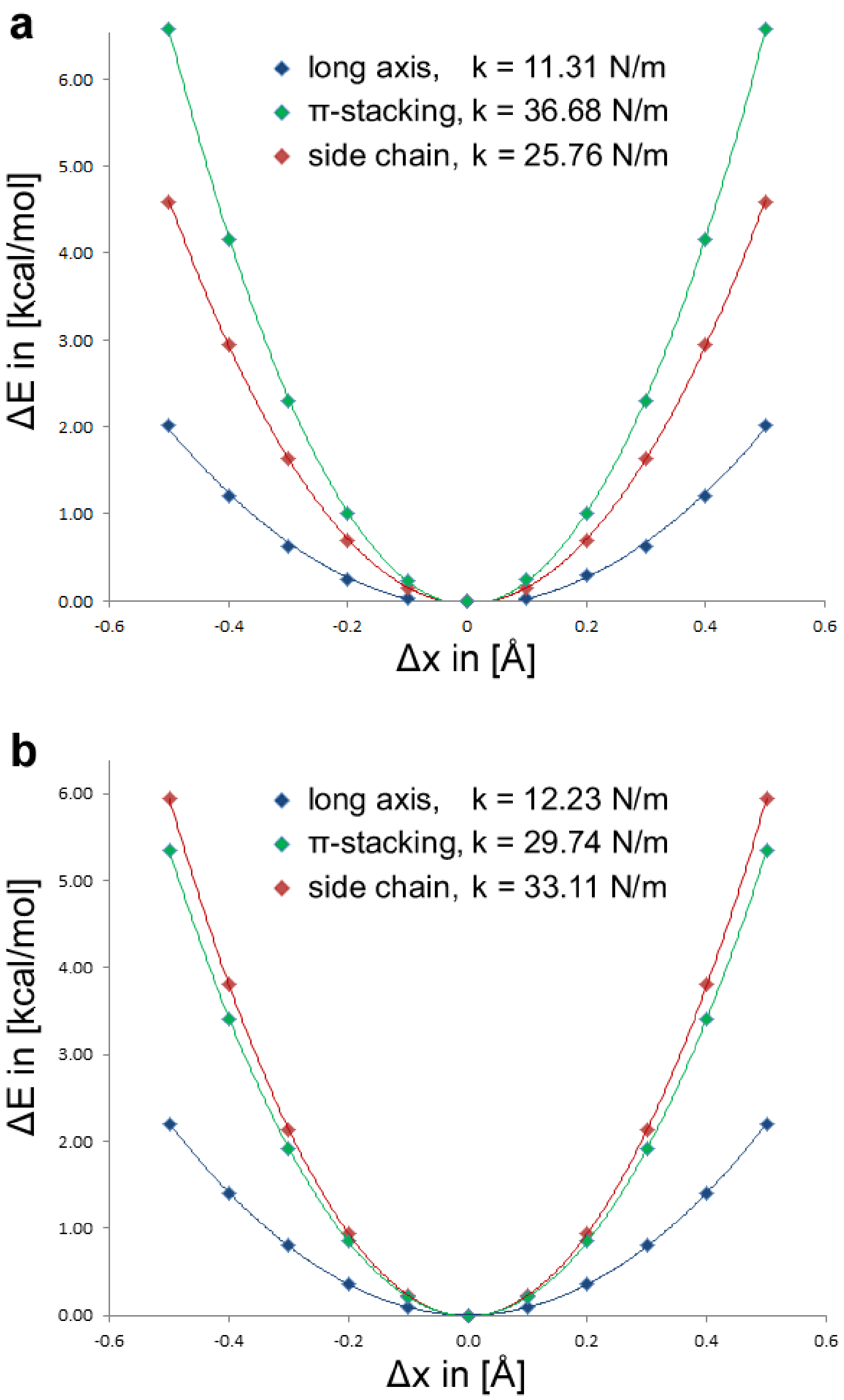


**Supplementary Figure 14:** Plot of the computed potential energy changes ΔE as a function of molecular displacement Δx for TIPS-P (a) and diF-TESADT (b). The force constants k were obtained from the polynomial fits by taking the second order derivative.

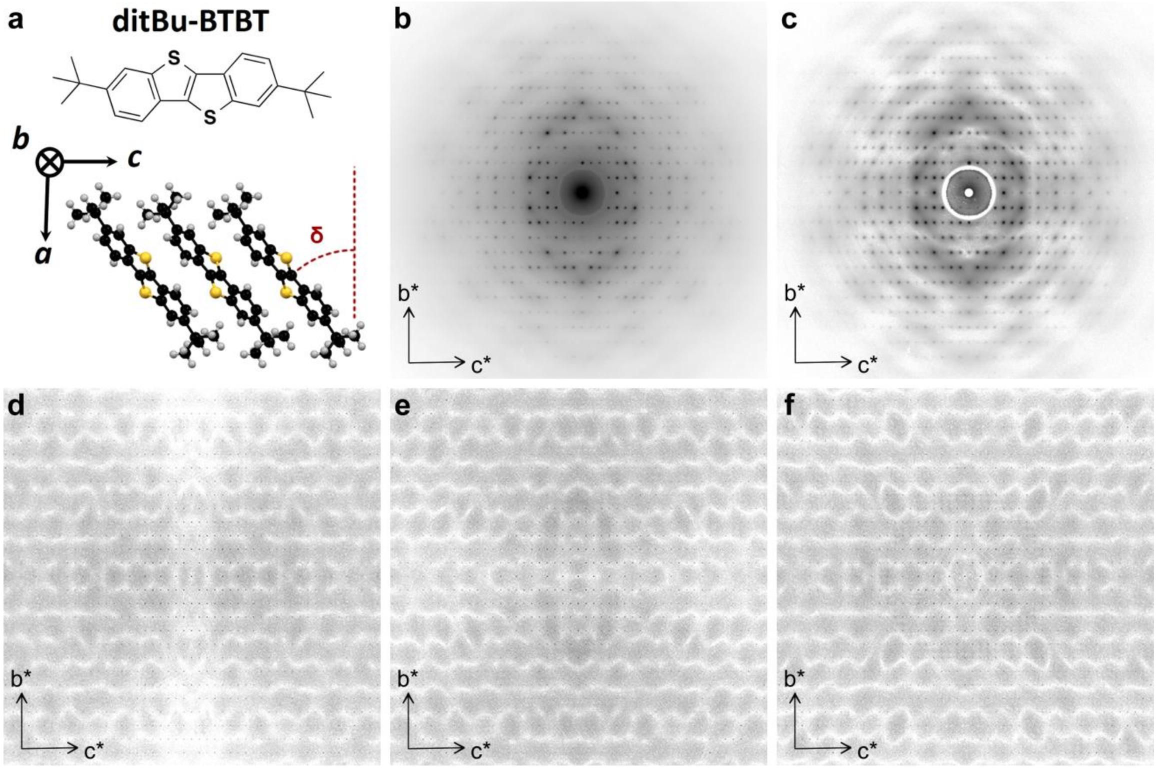


**Supplementary Figure 15:** (a) Molecular and crystal structure of ditBu-BTBT. (b) Experimental diffraction pattern of ditBu-BTBT presented in a logarithmic intensity scale to make the faint diffuse streaking visible. (c) Same diffraction pattern after background correction. (d,e,f) Simulated diffraction pattern with an amplitude of σ=0.03 Å in (d) long-axis, (e) short-axis and (f) π-π stacking direction.

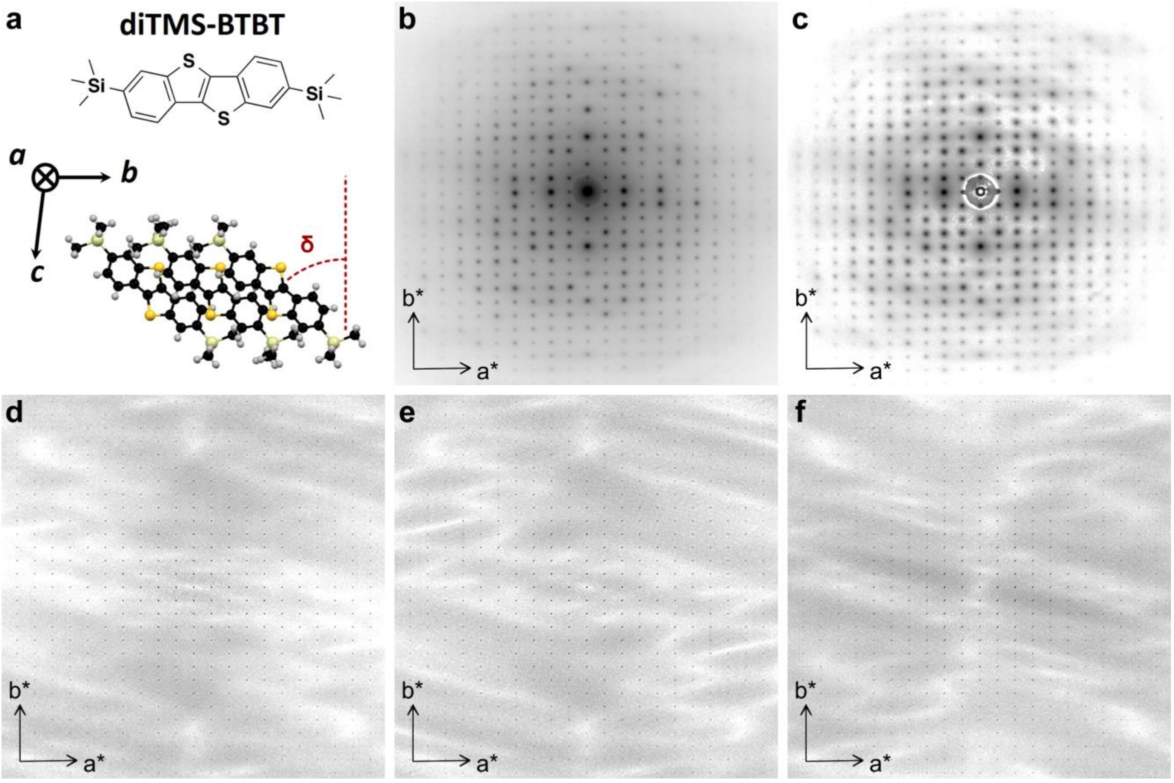


**Supplementary Figure 16:** (a) Molecular and crystal structure of diTMS-BTBT. (b) Experimental diffraction pattern of diTMS-BTBT presented in a logarithmic intensity scale to make the faint diffuse streaking visible. (c) Same diffraction pattern after background correction. (d,e,f) Simulated diffraction pattern with an amplitude of σ=0.03 Å in (d) long-axis, (e) short-axis and (f) π-π stacking direction.

| TIPS-P, in [Kcal/mole] | Equilibrium Structure (no displacement) | Displacement in long-axis direction (0.2 Å) | Displacement in π-π stacking direction (0.2 Å) | Displacement in side chain direction (0.2 Å) |
|---|---|---|---|---|
| Total Potential Energy | -237.88 | +0.30 | +1.00 | +0.69 |
| Intermolecular Energy | -1215.89 | +0.28 | +1.00 | +0.78 |
| Bond Stretching | 114.47 | -0.05 | -0.06 | -0.04 |
| Angle Bending | 196.05 | -0.23 | -1.15 | -0.81 |
| Stretch-Bend | -16.74 | 0.00 | -0.03 | -0.02 |
| Angle-Angle | -10.98 | -0.01 | -0.05 | -0.03 |
| Out-of-plane Bend | 0.15 | 0.00 | +0.02 | 0.00 |
| Torsional Angle | -617.09 | +0.09 | +0.62 | +0.27 |
| Stretch-Torsion | -0.84 | -0.01 | -0.02 | -0.02 |
| Van-der-Waals | -89.90 | +0.51 | +1.68 | +1.35 |
| Dipole-Dipole | 187.00 | 0.00 | 0.00 | -0.01 |
| Geometric Constraints | 0.00 | 0.00 | 0.00 | 0.00 |

**Supplementary Table 1:** Energetic components for TIPS-P and changes upon displacement of the conjugated core in [Kcal/mole].

| diF-TESADT, in [Kcal/mole] | Equilibrium Structure (no displacement) | Displacement in long-axis direction (0.2 Å) | Displacement in π-π stacking direction (0.2 Å) | Displacement in side chain direction (0.2 Å) |
|---|---|---|---|---|
| Total Potential Energy | 33.70 | +0.35 | +0.85 | +0.94 |
| Intermolecular Energy | -1245.53 | +0.26 | +0.41 | +0.61 |
| Bond Stretching | 71.43 | 0.00 | 0.00 | +0.03 |
| Angle Bending | 768.28 | +0.04 | +0.02 | +0.14 |
| Stretch-Bend | -13.09 | -0.01 | -0.01 | 0.00 |
| Angle-Angle | 6.55 | 0.00 | 0.00 | 0.00 |
| Out-of-plane Bend | 0.05 | 0.00 | +0.01 | 0.00 |
| Torsional Angle | -8.79 | +0.01 | +0.41 | +0.08 |
| Stretch-Torsion | -0.20 | -0.01 | -0.01 | -0.01 |
| Van-der-Waals | -616.95 | +0.27 | +0.40 | +0.67 |
| Dipole-Dipole | -173.57 | +0.03 | +0.01 | +0.02 |
| Geometric Constraints | 0.00 | 0.00 | 0.00 | 0.00 |

**Supplementary Table 2:** Energetic components for diF-TESADT and changes upon displacement of the conjugated core in [Kcal/mole]

**Supplementary Note 1: TEM simulation and refinement**

Technical details about the simulation and refinement procedure have been published in [4]. The TEM simulation source code written in C++ has been made available online [5]. Note that a CUDA enabled graphic card is required to execute the simulation. The input files that were used for TIPS-P, diF-TESADT and C8-BTBT simulations are provided as examples. Provided is also a practical guide that describes how to simulate the diffuse scattering arising from thermal vibrations without the need to write a single line of source code.

The simulation computes the diffraction pattern based on a static supercell that includes the atomic displacements from a thermal vibration. Taking static snapshots of the thermally vibrating crystal lattice is justified by the experiment itself: In a TEM with an acceleration voltage of 300 keV as in this work (Philips CM30 TEM) the electrons are brought to approximately 80% the speed of light [6]. When such a relativistic electron passes through the crystal lattice of the organic thin-film that vibrates with typical frequencies of $10^{12}$ – $10^{13}$ Hz [7], the electron encounters a frozen snapshot of the vibrating crystal. A simulation of thermal vibrations via static displacements is, therefore, referred to as *frozen-phonon model* in the literature [8]. The experimental diffraction pattern is a superposition of all the different frozen crystal structures experienced by numerous electrons during TEM exposure time. The simulation works in a similar way and generates a number of static supercells, each large enough to incorporate thermal vibrations, and the corresponding diffraction patterns are calculated for each of them. Exactly as in the experimental setup, the final simulated pattern is the superposition of these individual patterns.

Each super cells typically incorporates 10,000 – 15,000 molecules (500,000 – 1,000,000 atoms). The simulation computes first the ideal crystal structure based on the published crystallographic data and then incorporates the thermal displacements which are randomly chosen from a Gaussian distribution with standard deviation σ. This magnitude is referred to as *amplitude*. The large super cell allows a statistically significant number of displacements to be incorporated. Computation for such a large structure has been made feasible in an appropriate amount of time by graphical processing unit (GPU) approaches [9–11]. Pushing the computation from to CPU on to the GPU allowed optimizing and parallelizing a large number of calculation steps, reducing simulation time significantly. On our system which runs a NVIDIA Quadro K600, a simulation of a full super cell takes between 1 – 30 minutes, depending on the size of the structure and the final diffraction pattern. The simulation itself is based on a *multislice* approach that follows the work of Kirkland [12]. Multislice means that the electron wave subsequently transmits through sub layers of the crystal structure. This allows to correctly account for dynamic effects (= multiple scattering events) during TEM simulation.

The simulated parallel incident electron beam results in sharp δ-function shaped Bragg reflections. The reflections in the experimental patterns appear broad in comparison which is an artefact of experimental detectors, usually referred to as *blooming effect*. We decided to not incorporate any graphical rendering that would change the simulation data in order to mimic this artefact, in particular as there is no advantage that would benefit the refinement procedure.

The refinement of the simulated data to match the experimental data is done in several steps. First, the simulation is run for a number of different sized crystals to determine the thickness of the experimental organic thin film. Here, atomic force microscopy measurements of organic films that

have been produced in the same thin-film batch are a good control of the simulation result and provide a first idea of the expected thickness range. The precise thickness of the experimentally investigated specimen is determined by analysis of the dynamical Bragg intensities. Thickness scans of all materials investigated in this study are shown in Supplementary Fig. 4-8. The second refinement step is to find a simplified model that is capable of describing the most dominant thermal vibrations in the crystal. This includes identifying the molecular fragments which exhibit the largest vibrational amplitudes, the nature of the vibration (e.g. translation, liberation; compare with Fig. 2c in the main text) and the direction in which these fragments predominantly vibrate (compare with Fig. 3 in the main text). The shape and characteristic of the diffuse features changes drastically if these assumptions are changed. A number of simulations are created during this refinement step and the best model is chosen by comparison with experimental data. After the nature and the direction of the vibrations have been identified, the final step is to refine the magnitude of the vibration amplitude. During each quantitative refinement the R2 residual is minimized [13].

$$R_2 = \frac{\sum_h \left| I_h^{obs} - K \cdot I_h^{calc} \right|}{\sum_h I_h^{obs}} \qquad (1)$$

$I^{obs}$ and $I^{calc}$ are the experimental and simulated intensities either of the reflections or the diffuse streaks, depending on the refinement step. K is a normalizing factor that is computed from the sums of the Bragg reflection intensities as following:

$$K = \frac{\sum_h I_h^{obs}}{\sum_h I_h^{calc}} \qquad (2)$$

The refinement error is calculated following the method published in ref. 14. Details on the refinement of TIPS-P have been published in ref. 4.

**Supplementary Note 2: Analysis of the energetic components and derivation of the force constants**

3x3x3 super cells of TIPS-P and diF-TESADT were analyzed based on the MM3 force field published in [15]. The size of the super cells was chosen to prevent the molecules from interacting with their image through the periodic boundary conditions. The super cells were constructed based on the crystal structures published in [16] and [17] for TIPS-P and diF-TESADT, respectively. Both structures could be experimentally confirmed via electron diffraction studies. In a first step the structures were allowed to energetically relax to find their most stable equilibrium position. A few bond stretching, angle bending and torsional parameters, mainly between the silicon and its surrounding atoms are missing in the standard MM3 description and had to be provided manually.

To investigate how intermolecular displacements affect the potential energy in the crystal two atoms of each conjugated core in the super cell were constrained (one carbon atom in the 2$^{nd}$ and 4$^{th}$ ring of the molecular core). The constrained atoms were used to dictate the position of each conjugated core within the structure. 26 of the 27 conjugated cores in the 3x3x3 super cell were constrained at their equilibrium position and the remaining molecule was displaced along either its long-axis, in π-π stacking direction or in direction of the molecule's side chains. Each structure was allowed to relax in its energetically most stable geometry afterwards. Constraining only two atoms rather than the full conjugated core allowed the residual atoms to relax in their most stable configuration. This prevents single atoms from being forced in an unnaturally close and energetically unstable geometry and, therefore, helps to ensure a more realistic study of the energetic components within the crystal. A breakdown of the potential energy into its components is shown in Supplementary Table 1 and 2 for TIPS-P and diF-TESADT, respectively. Each table shows the energetic components for the equilibrium structure and changes upon displacements of 0.2 Å in all three molecular directions.

Force constants k for displacements of the conjugated core were derived for all three molecular directions. Here, we displaced the conjugated core as described above in 0.1 Å steps around its equilibrium position. The change in potential energy ΔE as a function of displacement Δx can be fitted with a parabola, following $E = 1/2*k*x^2$ [18]. Supplementary Fig. 14 shows the computed energies plotted with a second order polynomial function for molecular displacements in long axis direction (blue), in side chain direction (red) and π-π stacking direction (green) for TIPS-P (a) and diF-TESADT (b).

**Supplementary Note 3: Stiffness and stretching of the side chains**

This paragraph provides a simple calculation that explains why the side chains of a molecule stabilize the conjugated core in direction of the side chains but only marginally perpendicular to that direction. It is assumed that the restoring force F is proportional to the expansion x of the side chains (Hooke's law), i.e. $F = -kx$ with k being the force constant of the side chain. From electron diffraction data it has been shown that the side chains do not follow the thermal displacements of the conjugated core (Supplementary Fig. 1). This result is supported by molecular dynamics simulation and justified with the strongly enmeshed hydrogen-rich side groups. Therefore, it is a justified approximation to consider the silicon atoms in materials like TIPS-P, TMTES-T, TESADT or diF-TESADT as rigid which is additionally supported by their larger mass and their large number of intramolecular bonded hydrocarbons. The C-C≡C-Si fragment of the side chain which is anchored at the silicon atom comprises a total length of 4.59 Å. We assume a typical amplitude of 0.15 Å for thermal vibrations in different directions.

Two scenarios are considered:

1. **The conjugated core vibrates in direction of the side chains:**
   In that case the expansion or contraction of the side chain is directly given by the amplitude of the vibration and the restoring force computes to F = -k * 0.15 Å with k being the force constant of the C-C≡C-Si fragment.
2. **The conjugated core vibrates perpendicular to the direction of the side chains, e.g. in direction of the π-π stacking or the long axis of the conjugated core:**
   In this scenario the resulting stretching of the side chain is significantly smaller. For an in-plane displacement of the conjugated core of 0.15 Å the C-C≡C-Si fragment needs to expand by only 0.00245 Å, resulting in a restoring force of F = -k * 0.00245 Å with k being the force constant of the C-C≡C-Si fragment.

The restoring force induced by the side chains on a displacement of the core in direction of the π-π stacking or along its long axis is therefore > 60 times smaller than for a displacement in direction of the side chain. This explains why the side chains are capable of supressing the vibrational amplitude in out-of-plane direction but allow for sufficient flexibility for the conjugated cores to perform large-amplitude, in-plane vibrations.

The force constants k for intramolecular bonds such as the C-C≡C-Si fragment is approximately 10-100 times stronger than the typical force constant of a van-der-Waals interaction [19]. The factor of 60 reduces the impact of the side chains on in-plane vibrations of the conjugated core, such as vibrations in π-π stacking or long axis direction, to the same order of magnitude than that of a single van-der-Waals interaction. Therefore, it is essential to consider van-der-Waals forces to explain why particular in-plane vibrations exhibit a small amplitude (large force constant k) while others have a more pronounced amplitude (small force constant k), given the large number of single van-der-Waals interactions between adjacent molecules.

**Supplementary Note 4: Diffraction analysis of other BTBT derivatives**

We examined the diffraction pattern of 2,7-di-tertbutyl[1]benzothieno[3,2-b][1]benzothiophene (ditBu-BTBT) and bis(trimethylsilyl)[1]benzothieno[3,2-b][1]benzothiophene (diTMS-BTBT) to further support the result that synthesizing the side chains to the long axis of the conjugated core reduces the amplitude of thermal vibrations in that direction. In both molecules the conjugated cores stack at an angle to the substrate normal (40.5° in ditBu-BTBT and 51.3° in diTMS-BTBT shown in Supplementary Fig. 15.a and 16.a, respectively). Diffraction patterns acquired with an incident electron beam parallel to the substrate normal should thus carry traces of diffuse scattering that arises from all three directions of the molecular core, i.e. both materials are suited to investigate the strength of intermolecular vibrations along all three directions of the conjugated core.

The diffuse intensity in both, ditBu-BTBT and diTMS-BTBT, diffraction patterns is very broad, similar to the diffuse features observed in C8-BTBT and C10-DNTT. This is a result of the larger variety of reciprocal inter-atomic distances that arises from the presence of the sulfur atoms in the conjugated cores. In ditBu-BTBT and diTMS-BTBT, however, this effect is enhanced as a result of the inclined angles. In the examined [001] crystal structure of diTMS-BTBT, the two molecules even stack at different angles in their unit cell which results in an additional smearing-out of the diffuse intensity; giving rise to the broadest diffuse intensity observed up-to-date.

Unfortunately, such broad features are virtually impossible to reliably distinguish from the general diffuse background. In addition, the refinement model is more complex with at least three displacement parameters that describe intermolecular vibration in all three directions of the conjugated core which makes a quantitative refinement procedure unfeasible based on the acquired diffraction data. However, it is still possible to draw insightful conclusions regarding the relative strength of the long-axis vibration compared to other vibrations:

In all materials investigated in Fig. 1 of the main text, namely TIPS-P, TESADT, diF-TESADT, TMTES-P and rubrene, it has been possible to identify dominant diffuse features that could be exclusively assigned to the long-axis vibration. And this despite the fact that the majority of the investigated diffraction patterns were oriented in a way that dominant vibrations in short-axis or π-π stacking direction would have been equally visible (Fig. 3 in the main text shows this for TIPS-P). This situation is different in the investigated BTBT derivatives. Here, comparison of the simulated diffraction pattern based on only one intermolecular vibration fails to explain the experimental diffraction data. In particular, it is obvious that incorporating only the long-axis vibration in the refinement model is insufficient to explain the experimentally observed data, i.e. the long-axis vibration is not anymore dominant in BTBT derivatives (compare Supplementary Fig. 15.c and e or Supplementary Fig. 16.c and f). Note that the missing intensity drop-off at the edges of the simulated patterns is a result of the simplified vibrational models that assume the side chains rigid as described in the main text.

**Supplementary Methods: Transistor Studies**

Powders of TIPS-P, TMTES-P, TESADT and diF-TESADT were dissolved in a 0.01 weight percent solution in tetralin and drop-casted onto a glass substrate with a thin-film of PSS that was kept at a temperature of 60 °C until all of the solvent evaporated. Subsequently, the single crystals were transferred via a floating technique onto a Si substrate with 300 nm of thermally grown $SiO_2$ that had been treated with OTS. Top gold contacts were evaporated using TEM grids that can be freely positioned on the crystals as shadow masks. The channel length was 20 µm; the channel width is given by the size of the contacted crystal and has been measured optically for each device.

The mobilities of our single-crystal TIPS-P devices are an order of magnitude below previously reported values that were measured in devices with spin-coated films of TIPS-P [20]. Our diffraction data shows that the structure of solution-grown single-crystal TIPS-P coincides with the structure reported in ref. 21. This allowed us to calculate the transfer integrals of single-crystal based on the method given in ref. 22. We found exceptionally low values for the in-plane coupling between adjacent TIPS-P molecules of 7 $cm^{-1}$, 69 $cm^{-1}$ and 83 $cm^{-1}$ which is significantly lower than the calculated transfer integrals in TMTES-P and rubrene presented in the main text. We assume that the combination of small transfer integrals and high electron-phonon couplings [4] is the reason why drop-casted, single crystals of TIPS-P perform badly.

Rubrene was purchased from Sigma-Aldrich and used without further purification. Single crystals were grown in a MTI OTF 1200X furnace by the physical vapour transport method [23] at atmospheric pressure in a high-purity argon flow of 100 sccm. Typical growth conditions were: 310 °C at the sublimation zone and a growth duration of approximately 75 min.  Crystals varied in thickness from hundreds of nanometres to several micrometres.

Field effect transistors of C8-BTBT were also fabricated in a bottom-gate, top-contact architecture. The bilayer dielectric consists of 200 nm of atomic layer deposited $Al_2O_3$ and a 70 nm thick film of spin-coated CYTOP. C8-BTBT has been thermally evaporated with a thickness of 50 nm. The source drain contacts consist of 7 nm of $MnO_3$ followed by 25 nm of gold. The channel length measures 500 µm, the channel width 1 mm. We want to emphasis the polycrystalline nature of the evaporated C8-BTBT films and that we have not been able to grow high-quality, sufficiently thin crystals of C8-BTBT that were suitable for device fabrication. However, we were able to reproduce the wedge-casting technique reported in ref. 18 and achieved mobilities up to 9 $cm^2/Vs$ in single crystal C10-DNTT devices; in good agreement with values reported in literature [24, 25].

**Supplementary References**


1. Gonzalo Rincon Llorente, Marie-Beatrice Dufourg-Madec, David J. Crouch, Robin G. Pritchard, Simon Ogierc and Stephen G. Yeates. “High performance, acene-based organic thin film transistors”. Chemical communications, 21, Pages 3059-61, 2009
2. Stephen G. Yeates. Citation of TMTES-P crystal structure from the Cambridge Structural Database: Reference CCDC 1400822
3. Gaurav Giri, Eric Verploegen, Stefan C. B. Mannsfeld, Sule Atahan-Evrenk, Do Hwan Kim, Sang Yoon Lee, Hector A. Becerril, Alan Aspuru-Guzik, Michael F. Toney and Zhenan Bao. Tuning charge transport in solution-sheared organic semiconductors using lattice strain. Nature, 480, Pages 504-8, 2011.
4. A.S. Eggeman, S. Illig, A. Troisi, H. Sirringhaus, P. Midgely. “Measurement of molecular motion in organic semiconductors by thermal diffuse electron scattering”. Nat. Mater., 12, Pages 1045-1049, 2013
5. S. Illig. Thermal Diffuse Electron Scattering with CUDA. GitHub repository, 2015. URL: https://github.com/Steffen-Illig
6. David B. Williams and Barry C. Carter. “Transmission Electron Microscopy - A Textbook for Materials Science”. Springer, Page 14, ISBN 978-0-387-76501-3
7. Ivan Pelant and Jan Valenta. Luminescence Spectroscopy of Semiconductors. Oxford University Press, 2012, page 115. ISBN: 978-0-19-958833-6.
8. Loane, R. F., Xu, P. & Silcox, J. Thermal vibrations in convergent-beam electron diffraction. Acta Crystallogr. A 47, 267–278, 1991.
9. Lindholm, E., Nickolls, J., Oberman, S.&Montrym, J. NVIDIA Tesla: A unified graphics and computing architecture. IEEE Micro 28, 39–55, 2008.
10. Tickner, J. Monte Carlo simulation of X-ray and gamma-ray photon transport on a graphics-processing unit. Comput. Phys. Commun. 181, 1821–1832, 2010.
11. Dwyer, C. Simulation of scanning transmission electron microscope images on desktop computers. Ultramicroscopy 110, 195–198, 2010.
12. Kirkland, E. J. Advanced Computing in Electron Microscopy, Plenum, 1998.
13. Giacovazzo, C. et al. Fundamentals of Crystallography, Oxford Univ. Press, 2002.
14. R. Vincent, D. M. Bird and J. W. Steeds. Structure of AuGeAs determined by converget-beam electron diffraction II. Refinement of structural parameters. Philosophical Magazine A, 1984, Vol 50, No 6, 765-786, 1984
15. Jenn-Huei Lii, Norman L Allinger, J. Am. Chem. Soc., Molecular Mechanics. The MM3 force field for hydrocarbons, 1989.
16. Anthony, J. E., Brooks, J. S., Eaton, D. L. & Parkin, S. R. Functionalized pentacene: Improved electronic properties from control of solid-state order. J. Am. Chem. Soc. 123, 9482–9483, 2001.
17. Sankar Subramanian, Sung Kyu Park, Sean R. Parkin, Vitaly Podzorov, Thomas N. Jackson, and John E. Anthony. "Chromophore Fluorination Enhances Crystallization and Stability of Soluble Anthradithiophene Semiconductors". J. Am. Chem. Soc, Vol. 130, 2706-7, 2008.
18. Jain, Mahesh C. Fundamental forces and laws: a brief review. Textbook of Engineering Physics, Part 1, Page 11, ISBN 9788120338623.

19. Linnett J. W., Trans. Faraday Soc., The force constants of some carbon-carbon bonds, 1941.
20. Jui-Fen Chang et al. Hall-Effect Measurements Probing the Degree of Charge-Carrier Delocalization in Solution-Processed Crystalline Molecular Semiconductors, PhysRevLett. 107, 066601, 2011.
21. John E. Anthony, James S. Brooks, David L. Eaton, Sean R. Parkin. "Functionalized Pentacene: Improved Electronic Properties from Control of Solid-State Order". Journal of the American Chemical Society, Vol. 123, No. 38, Pages 9482-9483, 2001.
22. Troisi, A., Orlandi, G. & Anthony, J. E. Electronic interactions and thermal disorder in molecular crystals containing cofacial pentacene units. Chem. Mater. 17, 5024–5031, 2005.
23. Hui Jiang and Christian Kloc. "Single-crystal growth of organic semiconductors". MRS Bulletin, Vol. 38, Issue 01, 2013.
24. T. Uemura a, K. Nakayamaa, Y. Hirose, J. Soeda, M. Unoa, W. Li, M. Yamagishi, Y. Okadaa, J. Takeya. "Band-like transport in solution-crystallized organic transistors". Current Applied Physics, 1-5, 2012.
25. Takafumi Uemura, Cedric Rolin, Tung-Huei Ke, Pavlo Fesenko, Jan Genoe, Paul Heremans, Jun Takeya. On the Extraction of Charge Carrier Mobility in High-Mobility Organic Transistors. Adv. Mater. 2015.
